# The Shared Pathological Niche in Pulmonary Fibrosis: From Aberrant Epithelial Repair to ECM Remodeling

**DOI:** 10.3390/biomedicines14102265

**Published:** 2026-10-07

**Authors:** Lu Yang, Yuting Huang, Andong Liu, Yuanmei Yan, Bo Liu, Xianglin Yuan

**Affiliations:** 1 Department of Oncology, Tongji Hospital, Tongji Medical College, Huazhong University of Science and Technology, Wuhan 430030, China; luciayang0611@163.com (L.Y.); hyt012345678@163.com (Y.H.); u202010378@hust.edu.cn (Y.Y.); 2 Department of Human Anatomy, School of Basic Medicine, Tongji Medical College, Huazhong University of Science and Technology, Wuhan 430030, China; liuandong@hust.edu.cn

**Keywords:** pulmonary fibrosis, EMT, macrophages, fibroblast activation, ECM

## Abstract

Pulmonary fibrosis is a frequent complication of abnormal tissue repair following lung injury by many causes. It can be found in various diseases and clinical conditions, such as idiopathic pulmonary fibrosis, cancer-treatment-induced lung injury, and other occupational, environmental, and autoimmune lung diseases. Though the initiating factors of these conditions are different, they have several biological mechanisms in common during disease progression, such as injury to and abnormal repair of alveolar epithelial and vascular endothelial cells, immune microenvironment remodeling, macrophage reprogramming, persistent activation of fibroblasts, excessive extracellular matrix deposition, and mechanically driven positive feedback loops. Recent single-cell and spatial multi-omics developments have changed the direction of pulmonary fibrosis research to concentrate on individual signaling pathways to obtain a wider perspective of interactions between cell types. These investigations have discovered significant cell populations, such as abnormal basal-like epithelial cells, SPP1^+^/TREM2^+^ profibrotic macrophages, and CTHRC1^+^ pathological fibroblasts. They have also uncovered new processes, including metabolic–epigenetic interactions, endothelial-to-mesenchymal transition, and macrophage-to-myofibroblast transition that give a new understanding to the pathogenesis of diseases. Pirfenidone and nintedanib may slow the progression of lung dysfunction but do not reverse already existing fibrosis. Thus, knowledge of the complicated cellular and molecular processes of pulmonary fibrosis is critical in determining new therapeutic targets and enhancing existing therapies. This review summarizes pathological characteristics, mechanisms and therapeutic breakthroughs with special emphasis on idiopathic pulmonary fibrosis and cancer-therapy-related lung damage and discusses emerging strategies targeting epithelial repair, endothelial barrier activity, macrophage activity, fibroblast activation, and extracellular matrix remodeling.

## 1. Introduction

Pulmonary fibrosis is not just a disease; it is a common pathological process that can occur in many lung conditions presenting fibrotic features. Different kinds of lung injury may trigger or promote its development, and its main features are described as destruction of the alveolar architecture, persistent activation of fibroblasts and myofibroblasts, and abnormal deposition of the extracellular matrix (ECM) [1,2,3,4,5]. As fibrosis progresses, scar-like fibrotic tissue gradually remodels and replaces normal alveolar structures, thus resulting in reduced lung compliance and impaired gas exchange. Patients may present with dyspnea, dry cough, reduced exercise tolerance, hypoxemia, and acute exacerbations; eventually they may progress to respiratory failure and even death [1,2,3,5].

Idiopathic pulmonary fibrosis (IPF) is one of the representative fibrotic lung diseases. IPF mainly occurs in middle-aged and older adults. Its cause is still not fully understood. IPF is characterized by an insidious onset, a progressive course, and a poor prognosis. Upon radiological and histopathological examination, it usually presents with a usual interstitial pneumonia (UIP) pattern [1,2,3]. After the concept of progressive pulmonary fibrosis (PPF) was proposed, the clinical understanding of fibrotic lung disease expanded beyond IPF to include multiple fibrotic interstitial lung diseases (ILDs) that share a progressive phenotype [1,5]. Meanwhile, treatment-related lung injury and its fibrotic progression are becoming significant clinical concerns in oncology. Thoracic radiotherapy, cytotoxic agents such as bleomycin, molecularly targeted agents such as epidermal growth factor receptor–tyrosine kinase inhibitors (EGFR-TKIs) or anaplastic lymphoma kinase–tyrosine kinase inhibitors (ALK-TKIs), immune checkpoint inhibitors (ICIs), and antibody–drug conjugates (ADCs) may all cause lung injury [6,7,8,9,10,11,12,13,14,15,16,17]. In some patients, acute or subacute pneumonitis may be followed by persistent interstitial abnormalities, volume loss, traction bronchiectasis, and fibrotic changes, suggesting that, although the initial cause may differ, they can converge in common pathways of aberrant repair and fibrosis.

Traditionally, pulmonary fibrosis was regarded as post-inflammatory scarring. However, anti-inflammatory or immunosuppressive therapies have failed to provide a clinical benefit in IPF and have even caused harm [18]. These findings suggest that fibrosis is not simply decided by how strong the inflammation is, but rather by whether tissue repair after injury can restore homeostasis [4,5,18,19]. Normal repair after lung injury depends on the coordinated interaction of alveolar epithelial regeneration, resolution of inflammation, macrophage efferocytosis, restoration of the vascular barrier, and dynamic ECM remodeling. If injury continues or repair becomes dysregulated, damaged epithelial and endothelial cells release damage-associated molecular patterns (DAMPs), chemokines, and profibrotic mediators; then, these factors drive remodeling of the immune microenvironment and macrophage reprogramming. Paracrine signals, ECM stiffening, and mechanical forces influence fibroblasts, causing them to become activated and differentiate into myofibroblasts [4,19,20,21,22,23]. These cells deposit collagen, cross-link ECM, and stiffen tissue, activating latent transforming growth factor-β (TGF-β); they then establish a self-sustaining fibrotic niche that can lead to progressive lung tissue destruction [4,19,24,25,26]. Although IPF, PPF, and tumor treatment-related lung injury have different triggers and early inflammatory responses, their fibrotic processes share several pathological features, such as epithelial and endothelial injury, immune dysregulation, macrophage reprogramming, fibroblast activation, and ECM remodeling. While nintedanib and pirfenidone slow the decline in forced vital capacity (FVC), they only delay fibrosis rather than reverse it, and some patients still continue to progress [27,28,29]. Therefore, it is essential to clarify the shared mechanisms and heterogeneity of pulmonary fibrosis under different injury backgrounds, because this can help identify new therapeutic targets and improve current treatments; this review aims to summarize the key pathogenic mechanisms and research advances in pulmonary fibrosis.

## 2. Methods

### 2.1. The Literature Search Strategy

This article is a narrative review informed by a structured literature search. A comprehensive search was performed in PubMed (last search: September 2026) using a single search string that combined disease-related terms—covering idiopathic pulmonary fibrosis, pulmonary fibrosis, interstitial lung disease, usual interstitial pneumonia, radiation-induced lung injury, drug-induced pneumonitis, hypersensitivity pneumonitis, silicosis and other pneumoconioses, connective tissue disease-associated interstitial lung disease (systemic sclerosis and rheumatoid arthritis), as well as post-infectious (post-COVID-19) pulmonary fibrosis—with thematic terms covering single-cell and spatial transcriptomic technologies, key cell types and pathogenic mechanisms (including macrophages, neutrophils, fibroblasts and myofibroblasts, epithelial and endothelial cells, TGF-β signaling, extracellular matrix remodeling, cellular senescence, glycolysis, and epigenetic regulation), and clinical study design with emphasis on the most recent five years (2021–2026), but without strict restriction on publication year. The full search string is provided in Supplementary Table S1. The reference lists of retrieved articles were manually screened to identify additional relevant studies, and forward citation tracking was applied to key publications.

### 2.2. Eligibility Criteria and Study Selection

Titles and abstracts of retrieved records were screened for relevance to the pathogenesis, progression, or treatment of pulmonary fibrosis. Studies were eligible if they were peer-reviewed articles published in English focused on pulmonary fibrosis. Original research articles constituted the primary basis of evidence synthesis; review articles and guidelines were cited solely for background reference. Conference abstracts, unrefereed preprints, and articles lacking original data were excluded.

### 2.3. Study Classification and Data Synthesis

The included original studies were classified during full-text screening, according to their experimental design and data type, as single-cell transcriptomic studies, spatial transcriptomic studies, animal model studies (predominantly bleomycin- and radiation-induced models), or clinical studies. Findings were synthesized narratively and organized by cell type and pathogenic mechanism, including alveolar epithelial cells, macrophages, neutrophils, fibroblasts, endothelial cells, and the extracellular matrix, with emphasis on how single-cell and spatial transcriptomic technologies have advanced the understanding of cellular heterogeneity and intercellular crosstalk in pulmonary fibrosis.

## 3. IPF, Tumor Treatment-Related Pulmonary Fibrosis, and Others

### 3.1. IPF

IPF is a chronic, progressive, fibrosing interstitial pneumonia of unknown cause, occurring mainly in middle-aged and older adults. It is characterized by progressive dyspnea and a decline in lung function [1]. Its development is closely associated with cellular senescence, genetic susceptibility, telomere maintenance dysfunction, MUC5B (mucin 5B) promoter variants [30,31,32,33,34], cigarette smoking, air pollution, occupational exposure, viral infection, and gastroesophageal reflux-related microaspiration [4,5]. These factors can disrupt alveolar epithelial homeostasis, increase susceptibility to microinjury, and impair regenerative capacity. Lung tissue is therefore predisposed to abnormal repair and fibrotic remodeling after persistent or recurrent injury. Clinically, patients present with exertional dyspnea and a dry cough. Pulmonary function testing demonstrates restrictive ventilatory impairment, accompanied by a decline in the diffusing capacity for carbon monoxide (DLCO) [1,2]. High-resolution computed tomography (HRCT) plays a central role in the diagnosis of IPF. In the appropriate clinical context, typical or probable UIP patterns can support the diagnosis of IPF. The typical UIP pattern is characterized by reticulation in the bilateral basal and subpleural regions, with a basal and subpleural predominance, with or without traction bronchiectasis and honeycombing, and by the absence of imaging features that clearly suggest an alternative diagnosis [2,3].

Pathologically, UIP is characterized by marked temporal and spatial heterogeneity. Relatively normal lung tissue, fibrotic areas of varying stages, fibroblast foci, and honeycombing changes may coexist within the same lung specimen [2,4]. Among these features, fibroblast foci are generally considered key sites of active fibrosis. Within these foci, aberrant alveolar epithelial cells, macrophages, activated fibroblasts and myofibroblasts, and a stiffened ECM interact to maintain a profibrotic microenvironment and drive disease progression [4,19,20,21,22,24,25,26,35].

In recent years, single-cell sequencing studies have further transformed our understanding of the cellular pathological mechanisms of IPF. Adams et al. performed single-cell RNA sequencing on lungs from 32 IPF patients, 18 patients with chronic obstructive pulmonary disease (COPD), and 28 controls, and first identified an IPF-specific aberrant basaloid cell population (TP63^+^/KRT17^+^/SOX9^+^/COL1A1^+^/CDH2^+^, KRT5^−^/KRT15^−^), localized to the edge of myofibroblast foci; they also found that peribronchial vascular endothelial cells (COL15A1^+^) ectopically expanded into the distal lung parenchyma [36]. Habermann et al., analyzing 20 pulmonary fibrosis (PF) and 10 control lungs, identified KRT5^−^/KRT17^+^ ECM-producing epithelial cells (COL1A1^+^/FN1^+^/SOX9^+^/MMP7^+^/p16^+^) and fibroblast subtypes with distinct spatial distributions (ACTA2^+^ myofibroblasts, HAS1hi fibroblasts, and PLIN2^+^ fibroblasts) [20]. Together, these two studies revealed a key mechanism by which aberrant epithelial and mesenchymal cells cooperate to drive fibrosis in IPF. Meanwhile, Reyfman et al. identified a novel subset of profibrotic alveolar macrophages highly expressing secreted phosphoprotein 1 (SPP1) [37]. Another study confirmed that SPP1^+^/MERTK^+^ macrophages greatly increased in number and expressed more SPP1 in the fibrotic lower lobes of IPF, and these cells were closely linked to myofibroblast activation [38]. Then a recent study used GFP reporter mice combined with single-cell sequencing to systematically map out the atlas of lung collagen-producing cells, and this work questioned the old idea of a “single source of myofibroblasts” and showed that alveolar, adventitial, and peribronchial fibroblasts in normal lungs have differences in location. They also identified CTHRC1^+^ pathological fibroblasts that appear only in fibrotic lungs, and these cells express the highest levels of collagen and ECM genes. They also possess strong migratory and lung-homing capabilities, and are localized to the core of fibroblast foci; they are found in both human IPF and scleroderma lungs, providing a novel target for antifibrotic therapy [39]. Together, these studies build a multidimensional cellular pathological atlas of IPF and show that it is a complex disease where multiple cell lineages act together abnormally, laying the foundation for cell-type-specific interventions.

### 3.2. RIPF

Thoracic radiotherapy is an important treatment method for thoracic cancers like lung cancer and esophageal cancer, but normal lung tissue can be easily damaged by ionizing radiation, and this makes it likely that radiation-induced lung injury (RILI) will develop [6,7]. RILI is an umbrella term that encompasses two clinically recognized phases: early radiation pneumonitis (RP) and late radiation-induced pulmonary fibrosis (RIPF). These two phases are pathophysiologically linked, but the progression is not strictly linear, as RP may resolve without fibrosis or be delayed by years [6,7]. Ionizing radiation can directly cause DNA damage, mitochondrial dysfunction, oxidative stress, and hypoxia, and these effects injure alveolar epithelial cells and vascular endothelial cells [6,7,40,41]. Damaged cells release inflammatory factors and chemokines, which promotes recruitment of inflammatory cells and interferes with regeneration of normal tissue [6,7,19,42]. What distinguishes RIPF from fibrosis of other etiologies is the persistent radiation-specific footprint and microenvironmental remodeling. Residual DNA damage sustains chronic cellular senescence and SASP secretion (TGF-β1, IL-6) long after exposure, and senolytic clearance of these senescent cells can reverse established fibrosis in preclinical models [43]. Irradiation drives resident lung mesenchymal cells toward a myofibroblast phenotype, in which upregulated αv integrins locally activate latent TGF-β1 stored in the extracellular matrix, sustaining fibroblast activation independently of TGF-β transcriptional upregulation [44]. Endothelial cells are similarly marked by radiation, undergoing apoptosis and loss of vascular integrity within the irradiated field [40]. Single-cell transcriptomic profiling of irradiated lungs has further shown that injured endothelial cells actively recruit immune cells through ligand–receptor interactions rather than serving as passive bystanders, underscoring the coordinated remodeling of the vascular–immune compartment in RILI [45]. In addition, irradiation induces monocyte-derived macrophage infiltration in the irradiated lung, where macrophage polarization evolves over the course of RILI from an early inflammatory M1 state toward a chronic M2 phenotype that is reinforced by reciprocal signaling with injured endothelium [46,47]. These M2 macrophages, together with direct irradiation itself, activate Src family kinases (SRC and YES1) in epithelial cells, driving myofibroblast activation independently of SMAD2 phosphorylation. Importantly, this pathway is engaged even under a clinically relevant fractionation of 2 Gy*10 fractions. Pharmacological blockade of SFKs with NXP900 from the day of irradiation attenuated collagen deposition in vivo, whereas the same inhibitor showed no efficacy in late-stage bleomycin-induced fibrosis, indicating that this axis operates specifically in early radiation-induced remodeling [46]. When the injury becomes chronic as described above, profibrotic pathways such as TGF-β remain persistently activated, and this drives fibroblasts to turn into myofibroblasts and causes excessive ECM deposition [7,19,26]. Aberrant ECM remodeling and matrix stiffening further enhance fibroblast activation, forming a profibrotic positive feedback loop [7,24,25,35].

RP typically appears weeks to months after radiotherapy, and its manifestations include cough, dyspnea, low-grade fever, chest tightness, and ground-glass opacities or consolidation in the radiation field on imaging [6,48]. Some patients progress to a chronic fibrotic stage, which is characterized by lung volume loss in the irradiated field, linear scarring, traction bronchiectasis, pleural thickening, and architectural distortion [6,48]. A meta-analysis systematically summarized the relationship between lung dose–volume parameters and RP risk, and it confirmed that the mean lung dose (MLD) is the most reliable predictor under conventional fractionation [TD50 = 30.8 Gy, 95% CI: 28.7–33.9 Gy] [49]. They also recommend V20 (volume of lung receiving ≥ 20 Gy) ≤ 30–35% and MLD ≤ 20–23 Gy to limit the risk of RP to ≤20% in definitively treated patients with non-small-cell lung cancer (NSCLC) [49]. Similarly, another meta-analysis of 836 patients with NSCLC who underwent concurrent chemoradiotherapy, reported an overall symptomatic RP rate of 29.8%; each 1% increase in V20 was associated with a 3% increase in RP risk [49]. After adjusting for V20 and other factors, the carboplatin/paclitaxel regimen carried a 3.3-fold higher RP risk than the cisplatin/etoposide regimen (OR = 3.33); patients >65 years old receiving carboplatin/paclitaxel had an RP risk exceeding 50% [50]. Fatal RP (1.9%) was associated with a daily dose >2 Gy, high V20, and lower-lobe tumor location [50]. Thus, radiation-induced pulmonary fibrosis exhibits clear dose-spatial distribution characteristics, while also being influenced by host susceptibility, underlying lung disease, and combined treatment modalities [6,11,49,50]. Notably, the remodeled fibrotic lung is not merely a passive scar but may constitute a pro-tumorigenic microenvironment that facilitates local recurrence or metastasis after radiotherapy. This has led to treatment approaches that target the radiation-induced milieu. Inhibitors of Src family kinases such as NXP900 (NCT05873686) and dasatinib block radiation-induced tissue remodeling [46]. Bispecific antibodies simultaneously targeting TGF-β and PD-1/PD-L1 have demonstrated the ability to attenuate both peritumoral and radiation-induced pulmonary fibrosis while enhancing anti-tumor immunity when combined with radiotherapy [51,52]. Targeting the fibrotic microenvironment that drives tumor recurrence, these strategies may prevent fibrosis while enhancing oncologic outcomes following thoracic radiotherapy [46,51,52].

### 3.3. Drug-Induced Interstitial Lung Disease

Drug-induced interstitial lung disease (DI-ILD) is an important pulmonary adverse effect of multiple drug classes. Anti-tumor therapy-related DI-ILD includes cyto-toxic agents, molecular-targeted drugs, immune checkpoint inhibitors (ICIs), and antibody–drug conjugates (ADC). Its imaging and pathological manifestations are markedly heterogeneous, including organizing pneumonia (OP), nonspecific interstitial pneumonia (NSIP), hypersensitivity pneumonitis (HP)-like changes, diffuse alveolar damage (DAD), and mixed interstitial patterns [9,16].

Bleomycin can accumulate in lung tissue, and by inducing DNA damage, oxidative stress, and inflammatory cell infiltration, it initiates alveolitis and subsequent fibrotic responses; this serves as a classic model for post-DI-ILD fibrotic remodeling [8,53,54].

In patients with advanced NSCLC, the overall incidence of ILD increases to about 1.1% with EGFR-TKIs (gefitinib 1.53%, erlotinib 0.80%, afatinib 1.00%, and osimertinib retreatment 3.01%), which supports the fact that ILD has class-effect toxicity. And the risk may be increased 5–25 times the background level in patients with pre-existing ILD [10]. A meta-analysis showed that severe treatment-related pneumonitis (TRP) occurs more often with concurrent EGFR-TKI plus thoracic radiotherapy (4.9%) than with sequential therapy (0.4%), and the relative risk went up about 12-fold, which indicates that combined therapy is less tolerable than sequential therapy [11]. Similarly, a meta-analysis reported a pooled pneumonitis incidence of 2.14% with ALK inhibitors in patients with advanced NSCLC, with high-grade events occurring in 1.33% of patients [17]. In the FLAURA2 trial, the incidence of ILD/pneumonitis with first-line osimertinib plus chemotherapy in patients with EGFR-mutated advanced NSCLC was 3%, and no clear additional ILD risk was observed compared with osimertinib monotherapy, although grade 3 or higher adverse events were more frequent (64% vs. 27%) [55]. In the PROFILE 1007 study, the incidence of grade 3 or higher treatment-related ILD in the crizotinib group was approximately 1% (two fatal cases), which is comparable to that observed with EGFR-TKIs [56].

ICIs enhance anti-tumor immunity by relieving T-cell suppression. Checkpoint inhibitor pneumonitis (CIP) occurs in approximately 3–5% of patients in clinical trials, with rates reaching 7–13% in phase I trials of NSCLC, and this may be higher in real-world settings. Anti-programmed cell death protein 1 (PD-1) agents carry a greater risk than anti-programmed death-ligand 1 (PD-L1) agents, and combined ICI therapy or concurrent chemoradiotherapy can further increase the risk [15,16]. The median time to onset is approximately 2.8 months, and imaging manifestations are predominantly OP (65%), NSIP, HP, and acute interstitial pneumonia/acute respiratory distress syndrome (ARDS) [15,16]. A multicenter retrospective study involving 1826 patients showed that ICI-associated ILD occurred in approximately 3.5% of cases, with a median time to onset of 2.3 months [12]. CT most commonly revealed ground-glass opacities. Fatal ILD occurred in approximately 9.4% (all in NSCLC), and the 6-month overall survival rate was 58.1% [12]. Besides, the risk of ILD was significantly elevated with subsequent osimertinib therapy in patients recently treated with nivolumab (75% vs. 13%, *p* = 0.04) [57]. The TATTON study further confirmed the ILD incidence reached 22% in the osimertinib plus durvalumab arm, leading to discontinuation of that regimen, whereas no similar risk was observed with osimertinib plus selumetinib or savolitinib [58]. These findings indicate that the risk of ILD is significantly amplified when EGFR-TKIs are combined with or sequenced after ICI therapy, necessitating rigorous assessment of treatment intervals and enhanced monitoring [58].

ADCs, particularly trastuzumab deruxtecan (T-DXd), can cause ILD/pneumonitis of varying grades. A pooled analysis of nine T-DXd monotherapy phase I/II studies (*n* = 1150) revealed an overall incidence of drug-related ILD of 15.4%, of which 77.4% were grade 1–2, 2.2% were fatal, with a median time to onset of 5.4 months; 87% of events occurred within the first year. With earlier treatment lines and optimized monitoring, the incidence declined in subsequent studies (e.g., 10.5% in DESTINY-Breast03), with fewer severe events [13,14].

Overall, DI-ILD often begins as inflammatory injury. However, under conditions of persistent or recurrent damage, TGF-β activation, fibroblast/myofibroblast expansion, and ECM deposition can collectively drive the transition of lung tissue from reversible inflammation to irreversible fibrosis [19,25,26,35].

### 3.4. The Other

**Figure 1:**
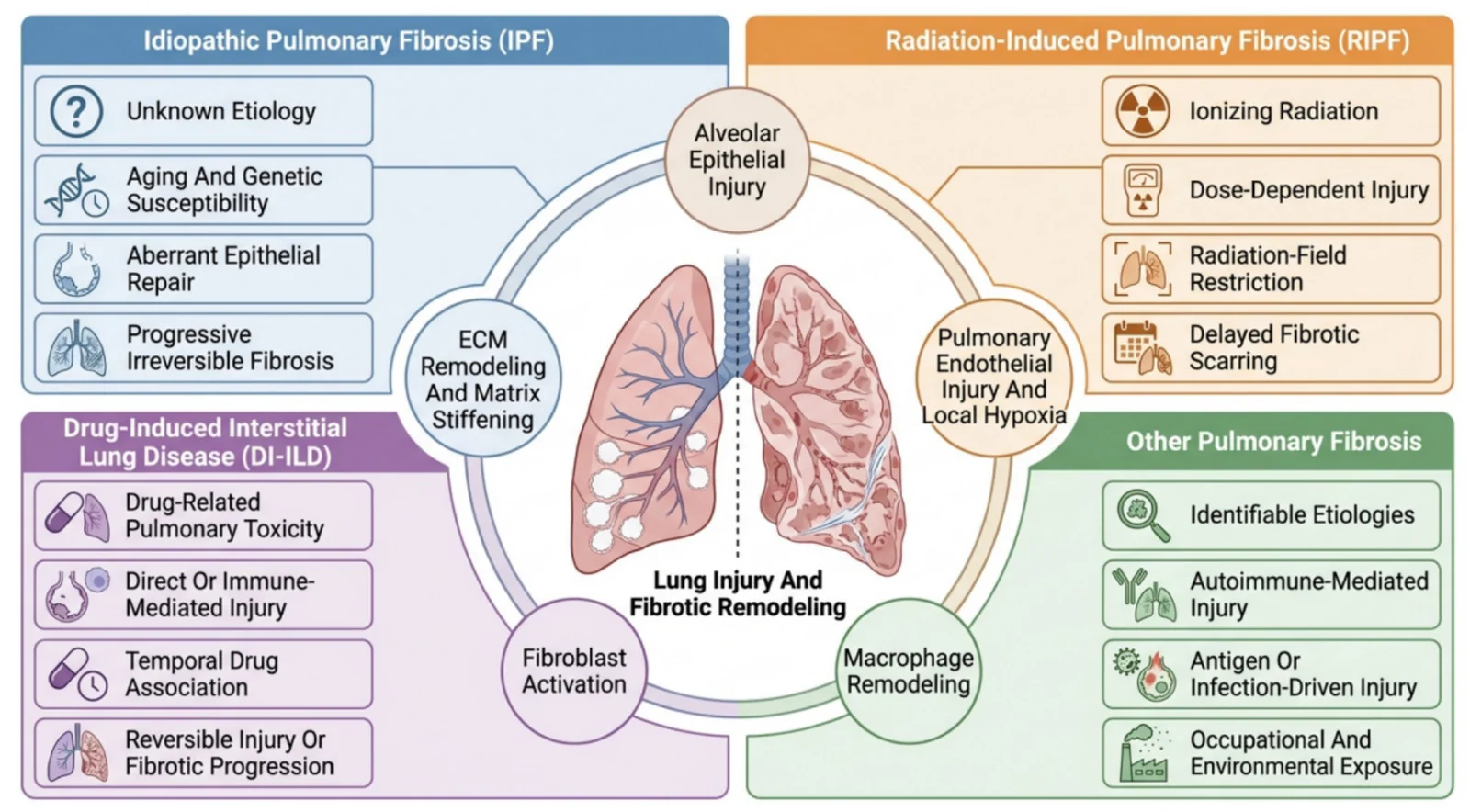
Schematic overview of the distinct etiological and clinical features of four pulmonary fibrosis contexts.

In addition to IPF and tumor-therapy-related pulmonary fibrosis, multiple diseases can also present with progressive pulmonary fibrosis phenotypes [1,5]. Connective tissue disease-associated ILD, particularly systemic sclerosis-associated ILD, involves autoimmune dysregulation, vascular and epithelial injury, and fibroblast activation [59]. Fibrotic hypersensitivity pneumonitis is mostly associated with chronic or repeated antigen exposure, and persistent antigenic stimulation can drive chronic inflammation and fibrotic interstitial remodeling [60,61]. Post-infectious pulmonary fibrosis has received increasing attention following severe viral pneumonia and acute respiratory distress syndrome (ARDS). After severe acute respiratory syndrome coronavirus 2 (SARS-CoV-2) infection, persistent interstitial abnormalities or fibrosis-like changes may occur, and the mechanisms may involve epithelial injury, inflammatory dysregulation, and activation of profibrotic pathways such as TGF-β [62,63,64,65]. Occupational- and environmental-exposure-related pulmonary fibrosis, such as silicosis- and air-pollution-related lung injury, is associated with particle-induced oxidative stress, macrophage activation, and NOD-like receptor family pyrin domain-containing 3 (NLRP3) inflammasome activation [66,67]. Exposure to air pollutants such as particulate matter with an aerodynamic diameter of ≤2.5 μm (PM2.5) is associated with the risk of IPF development, acute exacerbation, and lung function decline [68,69,70]. Although these diseases have diverse etiologies, they may share common mechanisms, including aberrant epithelial repair, immune microenvironment remodeling, fibroblast activation, ECM deposition, and matrix mechanical feedback. This helps place tumor-therapy-related pulmonary fibrosis within the broader spectrum of fibrotic ILD [19,20,21,22,24,25,26,35] (Figure 1).

## 4. Key Mechanisms in Pulmonary Fibrosis

**Figure 2:**
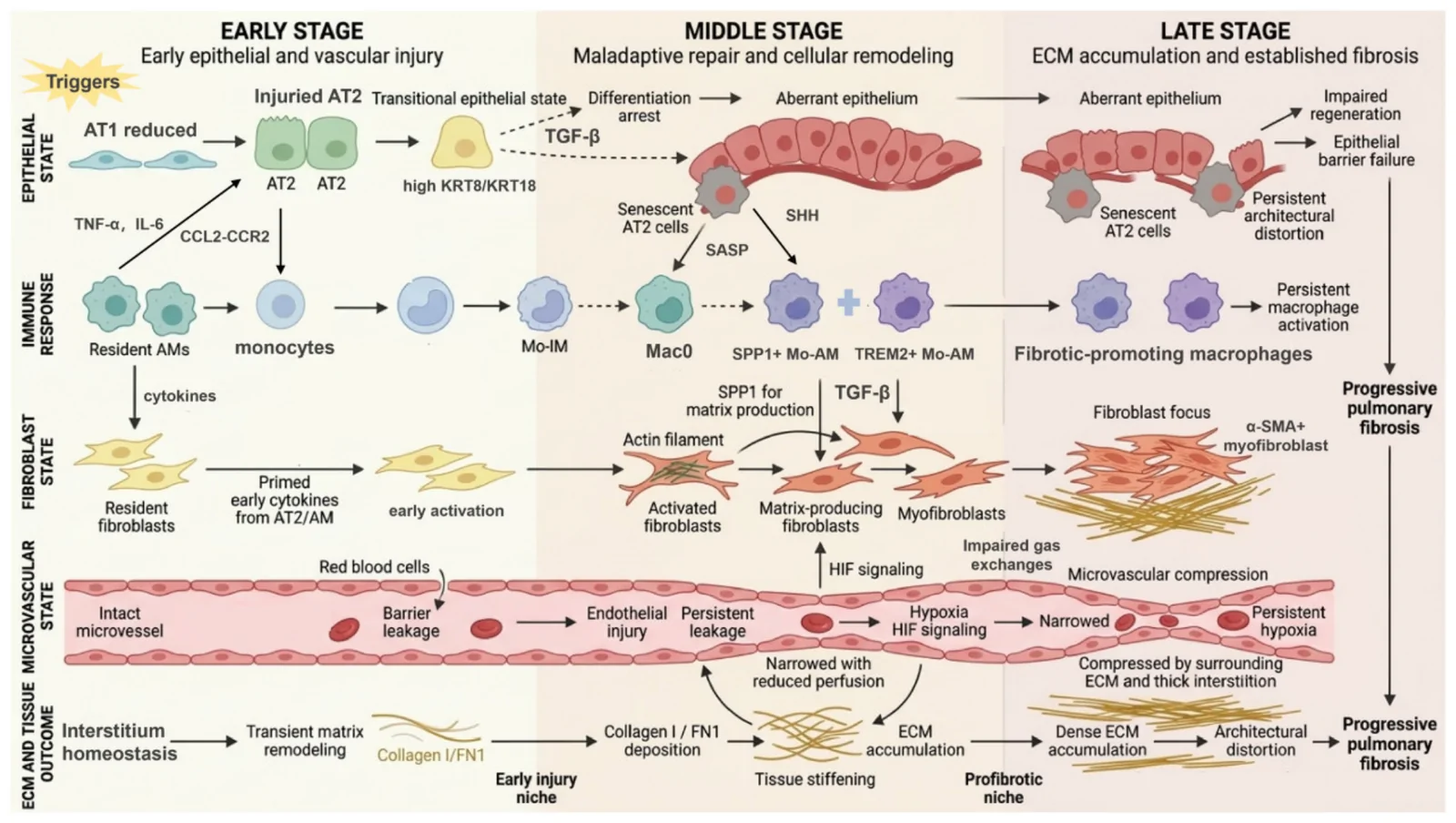
Integrated cellular mechanisms underlying the temporal progression of pulmonary fibrosis. Repetitive alveolar injury from diverse triggers disrupts tissue homeostasis and initiates aberrant repair. Epithelial injury impairs AT2-to-AT1 differentiation and promotes aberrant transitional epithelial states that release profibrotic mediators. Endothelial injury increases vascular permeability and capillary dysfunction, contributing to reduced perfusion and hypoxia. Recruited monocytes differentiate into profibrotic macrophages that suppress epithelial repair and promote fibroblast activation. Convergent signals from epithelial, endothelial, and immune dysfunction drive fibroblast activation and myofibroblast differentiation. Excessive ECM deposition and matrix stiffening establish self-amplifying feedback loops, ultimately causing architectural distortion and progressive fibrosis.

Pulmonary fibrosis begins with alveolar epithelial cell injury and defective repair, which leads to pulmonary endothelial cell injury and hypoxia. These insults activate macrophages as a key node linking injury and fibrosis, promoting fibroblast activation, ECM remodeling and stiffening of the mechanical environment (Figure 2).

### 4.1. Alveolar Epithelial Injury and Aberrant Repair

Damage to alveolar epithelial cells is an important initiating step in PF. The normal alveolar epithelium consists of alveolar type I (AT1) and alveolar type II (AT2) cells: AT1 cells occupy about 95% of the alveolar surface area, and are the main site of gas exchange [71]. AT2 cells produce pulmonary surfactant, can self-renew and differentiate into AT1 cells, and serve as progenitor cells upon injury to replace AT1 cells via proliferation and differentiation, thus preserving the structural integrity of the alveolus [71,72,73,74,75]. In the case of persistent injury or genetic predisposition, homeostatic dysregulation events can occur in AT2 cells, such as aberrant differentiation, senescence, metabolic reprogramming, epigenetic changes, and impaired intercellular communication. This implies that AT2 dysfunction is a key player in PF [75,76].

Early studies confirmed that AT2 cells have stem/progenitor cell properties. Through lineage tracing, it has been demonstrated that adult mouse AT2 cells can self-renew and differentiate into AT1 cells both during homeostasis and after injury, and this helps maintain alveolar architecture [74]. Building on these findings, another research used molecular marker screening, lineage tracing, and clonal analysis to reveal that alveolar development follows a functional transition from bipotent progenitors to mature AT2 stem cells [77]. During development, AT1 and AT2 cells arise independently from bipotent progenitors; after birth, these progenitors are exhausted, and mature AT2 cells become intermittently self-renewing bifunctional stem cells. In addition, AT1 injury can broadly activate their stem cell potential [77]. Subsequently, researchers further identified a Wnt-responsive subpopulation termed alveolar epithelial progenitors (AEPs), which make up about 20% of AT2 cells [78]. These cells maintain a stable lineage during homeostasis, but after injury they are selectively activated and undergo massive expansion to regenerate both AT2 and AT1 cells. This suggests that not all AT2 cells contribute equally to alveolar regeneration [78]. If AT2 cells do not expand and differentiate into mature AT1 cells in a timely manner, repair may shift from normal regeneration to aberrant repair, and fibrotic remodeling is promoted [71,75,76].

The alveolar epithelial injury triggers a repair program whereby AT1 and AT2 cells have different roles. Surfactant is produced by AT2 cells which are facultative progenitors. They multiply after injury and undergo two transient transitional states before differentiating into AT1 cells. The early state is characterized by high KRT8/KRT18 expression and activated TGF-β signaling, whereas the late state is characterized by KRT8/KRT18 downregulation, TGF-β deactivation, and full induction of AT1 markers. Knockdown of krt8 also affects differentiation of AT1 in cultured AT2 cells [79]. This program depends on maintenance signals from the mesenchymal niche, like FGF7-FGFR2 [80]. AT1 cells, in contrast, cover most of the alveolar surface for gas exchange and are normally quiescent [71]. Their reaction to injury is indirect because loss of AT1 stimulates regeneration through AT2 and the ability of AT1 cells to play a role in regeneration decreases with age [81].

AT2 cells face sustained injurious inputs in PF. Mutant surfactant proteins trigger the unfolded protein response, as shown in mice- and patient-derived cells carrying SFTPC C121G or C121Y [80], and elevated mechanical tension acts directly on alveolar stem cells to promote fibrosis [82]. These inputs convert transient repair signals into persistent ones. DNA-damage-induced TP53 activation drives AT2-lineage cells into a transitional state with a senescence-associated secretory phenotype [83]. Sustained YAP/TAZ activation in AT2 cells, as modeled by Stk3/4 deletion, prevents transitional cells from resolving and fibrosis from regressing after bleomycin injury [84], and profibrotic AT2-lineage cells establish an autocrine TGF-β positive feedback loop [83]. Together with chronic UPR signaling and AT2 senescence [80,85], these persistent signals arrest AT2 cells at the transitional stage. AT1 repair then fails as a downstream consequence, because stalled AT2-lineage cells no longer undergo differentiation toward AT1 [79,86], and the fibrosis they maintain stiffens the matrix and raises mechanical tension on the remaining alveolar stem cells [80,82,86]. Single-cell studies have provided important evidence for the aberrant epithelial states that result. KRT5^−^/KRT17^+^ pathological ECM-producing epithelial cells were identified in IPF lung tissue. These cells express COL1A1, FN1, MMP7, and CDKN2A, and their aberrant nature was noted by pseudotime analysis [20]. These cells were described as aberrant basal-like cells in other research, and they co-express basal markers such as TP63 and KRT17, mesenchymal markers (VIM, CDH2), and IPF-related molecules ITGAV/ITGB6. These cells are localized at the edge of myofibroblast foci, which suggests that epithelial cells may have acquired partial mesenchymal characteristics [36]. Subsequent studies further demonstrated that the persistent existence of this transitional state represents the core link in aberrant epithelial transformation. Then, an scRNA-seq in a bleomycin model found that airway and alveolar stem cell regeneration converged on a Krt8^+^ alveolar differentiation intermediate (ADI) state. This state was accompanied by p53/NF-κB activation, senescence programs, and profibrotic communication networks, and it persisted in human IPF [72]. Another research then identified the PATS from alveolar organoids and in vivo repair models, confirming that TP53 regulates its signature genes (Fn1, Ctgf, Cdkn1a, etc.), with PATS-like cells significantly enriched in IPF fibrotic regions [87]. Their reality is established independently of single-cell dissociation. In IPF sections, immunostaining shows KRT8-high transitional cells overlying fibroblastic foci and lining-dilated airspaces [79], and RNA in situ hybridization with SFTPC, and KRT17 probes identifies ABI1 and ABI2 cells in the same regions, with ABI2 cells found mostly adjacent to CTHRC1-high fibroblasts [88]. Spatial transcriptomics places aberrant basaloid cells within fibroblastic foci in independent cohorts [36,89,90]. The population is discrete and functional, as CD44-high cells sorted from fibrotic lungs retain an aberrant transcriptional program and drive fibroblasts toward a Cthrc1-positive fibrotic phenotype in culture [80]. Moreover, the state can be generated from normal human AT2 cells on demand, either by TGF-β combined with withdrawal of KGF/BMP4 maintenance signals [80] or by co-culture with pathologic mesenchyme, which carries them through ABI1 and ABI2 to KRT5-positive basal cells [88]. Finally, KRT8 variants are associated with IPF in a five-cohort genome-wide meta-analysis, linking these cells to human disease at the genetic level [86]. These populations differ from the KRT8-high transitional state of normal repair in their behavior. The transient state appears early after injury, gives rise to AT1 cells, and resolves as fibrosis regresses in mice acute lung injury [72,86]. Aberrant epithelial cells show the opposite behavior on each point. They persist in nonresolving lesions for at least nine weeks. Cultured aberrant basaloid cells fail to complete AT1 differentiation even under conditions in which normal transitional cells differentiate [86], and CD44-high cells from fibrotic lungs show impaired AT1 differentiation that is only partially rescued by co-culture with normal fibroblasts [80]. They additionally express p16/CDKN2A, a marker of permanent senescence [86]. The one apparent exception is that these cells complete transdifferentiation to basal cells within days in organoid culture [88]. This exception proves the point, because it shows that the trajectory is intact and that the block is imposed in vivo, where ABI2 and basal cells accumulate with increasing histologic severity instead of resolving [88] and where a basal-cell gene signature in bronchoalveolar lavage is associated with worse patient survival [91]. These populations are therefore stabilized pathological cell states maintained by the fibrotic niche.

It is worth noting that this aberrant epithelial state is related to the previously proposed concept of epithelial–mesenchymal transition (EMT), but it is also fundamentally different from it. Rock et al. utilized lineage tracing and found that in bleomycin-induced PF, labeled epithelial cells predominantly differentiated into alveolar epithelial type I (AEC1) cells rather than generating α-SMA^+^ myofibroblasts, which suggests that resident stromal cells, rather than epithelial cells, are the primary source of myofibroblasts [92]. Therefore, a statement more consistent with current understanding is that after injury, alveolar epithelial cells do not mainly transdifferentiate directly into fibroblasts through EMT; instead, they form aberrant transitional states during blocked regeneration, and these states show partial EMT-like or epithelial–mesenchymal hybrid phenotypes. This state still keeps epithelial attributes, but it is accompanied by blocked differentiation and maturation, decreased barrier function, and enhanced profibrotic paracrine signaling, and it represents a pathological epithelial plasticity state that connects epithelial injury with the formation of the fibrotic microenvironment.

Cellular senescence is closely related to this aberrant transitional state. Epithelial cells that undergo differentiation arrest are often accompanied by activation of the DNA damage response (DDR) and initiation of the senescence-associated secretory phenotype (SASP), and through this, intrinsic epithelial injury signals are converted into persistent drivers of the fibrotic microenvironment. Under persistent injury, senescence, or genetic susceptibility, AT2 cells may experience multi-level homeostatic imbalance, and they gradually enter senescence-related pathological states.

AT2 cell senescence is driven by systemic mechanisms such as telomere attrition and DNA damage. Mutations in telomerase reverse transcriptase (TERT) and the telomerase RNA component (TERC) have been established as genetic causes of familial IPF; detected in 8% of families, they reduce telomerase activity to 8–55% of wild-type levels, with carriers exhibiting approximately 30% shorter telomeres [33]. More importantly, even without detectable mutations, short telomeres themselves are a core risk factor for IPF, since 97% of sporadic patients have telomeres shorter than the age-matched median and 10% are below the first percentile; AT2 cell telomeres are shortened by approximately 60% [34]. Oxidative stress and telomere dysfunction can induce the DDR, and this activates the ATM/ATR-p53-p21 and p16 pathways, leading to cell cycle arrest and senescent phenotypes [93].

Mitochondrial dysfunction drives cellular senescence through progressive mechanisms. Bueno et al. found that PINK1 expression was reduced in AT2 cells from IPF patients, and impaired mitochondrial autophagy led to accumulation of malformed mitochondria and decreased respiratory chain activity [94]. It was subsequently confirmed that electron transport chain inhibition decreases the NAD^+^/NADH ratio, inducing mitochondrial dysfunction-associated senescence (MiDAS) via the AMPK-p53 axis. Under these conditions, p53 suppresses NF-κB activity. The IL-1-dependent inflammatory arm is blocked, and a distinct secretory phenotype that retains IL-10 and TNF-α is generated [95], which is closely related to senescence-associated tissue remodeling.

Endoplasmic reticulum (ER) proteostasis collapse is another core driver of senescence. In AT2 cells from old mice and IPF patients, GRP78 (glucose-regulated protein 78) is downregulated, while ER stress markers are elevated [96]. Borok et al. constructed a Grp78 knockout model, confirming that ER stress can directly induce AT2 cell senescence, apoptosis, and IPF-like fibrosis, with old mice being more susceptible to ER stress than young mice [96]. Burman et al. further revealed that the ER stress core effector molecule C/EBP homologous protein (CHOP) mediates ER stress-induced epithelial injury, as hypoxia induces CHOP expression through the dual pathways of the UPR (IRE1α/XBP1s and PERK/ATF4), and AEC apoptosis and fibrosis can be blocked by CHOP deficiency. This mechanism was validated by co-localization of CHOP with hypoxia markers in hyperplastic AT2 cells from IPF patients [97].

Senescent AT2 cells not only lose proliferative and differentiation potential but also transform into sources of profibrotic signals and acquire the SASP. The SASP is composed of cytokines, chemokines, matrix-remodeling proteases, and growth factors. Senescent AT2 cells continuously secrete IL-6, CCL2/MCP-1,SERPINE1/PAI-1, MMP10, MMP12, and other factors, amplifying local chronic inflammation through paracrine signaling and promoting fibroblast activation, myofibroblast differentiation, and abnormal ECM deposition and remodeling [32,75,76]. Therefore, selective clearance of pathological senescent cells or modulation of the SASP may represent an important strategy for intervening in AT2 cell senescence-associated PF.

### 4.2. Pulmonary Endothelial Injury and Local Hypoxia

Pulmonary endothelial cells exhibit marked heterogeneity. Schupp et al. integrated scRNA-seq data from six datasets comprising 73 healthy individuals; they identified >15,000 vascular and lymphatic endothelial cells, including six major types: lymphatic endothelial cells, arterial endothelial cells, venous endothelial cells (pulmonary venous and systemic venous ECs), and capillary endothelial cells, namely aCap and gCap [98,99]. aCap cells are highly specialized gas-exchange cells, and they are selectively distributed in the thin regions of the alveolar air–blood barrier, where they closely appose AT1 cells and form the core of the air–blood barrier. They possess a significantly larger surface area than gCap cells [98,99]. gCap cells are mainly located in the thick regions of the barrier, adjacent to interstitial cells and pericytes, and they take part in vascular tone regulation. Moreover, gCap cells show significant proliferative capacity after injury and can differentiate into aCap cells, which plays a critical role in capillary network repair and structural maintenance [98,99]. This heterogeneity allows the pulmonary microvasculature to carry out multiple functions at the same time, including gas exchange, barrier maintenance, coagulation–anticoagulation balance, immune trafficking, vascular tone regulation, and tissue repair.

Pulmonary capillary endothelial cells (PCECs) not only make up the alveolar–capillary barrier during normal lung injury repair, but they also actively control alveolar regeneration by producing organ-specific angiocrine factors. In PNX models, it has been shown that VEGFR2 and FGFR1 in PCECs are sequentially activated by PNX, which induces MMP14 expression. MMP14 then cleaves HB-EGF and a cryptic EGF-like domain in the laminin-5 γ2 chain, and this increases EGFR ligand bioavailability to drive expansion and differentiation of bronchoalveolar stem cells (BASCs) and AECIIs, initiating regenerative alveolarization [100,101]. Moreover, extensive ligand–receptor communication networks are present among pulmonary epithelial, endothelial, immune, and stromal cells, and these networks provide a structural interactive foundation for lung tissue homeostasis and post-injury regeneration [102,103]. In studies using conditional knockout mice, it has been confirmed that AT1-derived VEGFA specifically specifies and maintains Car4 ECs, which make up approximately 18% of lung endothelial cells. When this population is deleted, reduced alveolar capillaries and enlarged airspaces result, but endothelial proliferation or myofibroblasts are not affected, suggesting that alveolar morphogenesis is driven independently of myofibroblasts. On the other hand, when Car4 ECs are deleted, expression of neighboring AT1 markers decreases and differentiation is delayed, which indicates that the capillary endothelial niche supports alveolar epithelial homeostasis [104]. In PNX models, PCECs are activated via VEGFR2/FGFR1 and produce MMP14 to drive alveolar regeneration; when Vegfr2/Fgfr1 is deleted specifically in endothelial cells or MMP14 is blocked, repair is significantly impaired, and transplantation of wild-type PCECs can restore lung function, indicating that an intact pulmonary microvascular endothelial paracrine network is critical for post-injury alveolar regeneration [100]. Subsequent studies have shown that when BMP4 stimulates lung microvascular endothelial cells (LuMECs), thrombospondin-1 (TSP1) production is induced via the calcineurin/NFATc1 pathway, and this specifically promotes BASC differentiation into alveolar epithelial cells, which is essential for alveolar regeneration in bleomycin injury models [105].

However, under conditions of persistent injury, endothelial cell injury and endothelial state remodeling are increasingly recognized as important components of the profibrotic pathological niche. Endothelial cells are not merely passive victims of fibrosis but undergo systematic remodeling of cell proportions, subpopulation composition, and functional states, actively participating in and driving fibrosis. scRNA-seq has revealed that the ratio of aCap to gCap cells is significantly decreased in IPF lungs, whereas COL15A1^+^ venous endothelial cells are normally restricted to proximal bronchi ectopically expanded into distal lung parenchyma (comprising 54% of all endothelial cells versus 8.9% in controls), with immunohistochemistry confirming their distribution within fibrotic lesions [36]. This suggests that endothelial subpopulations with gas-exchange and vascular homeostatic functions are reduced in number, and this reduction is accompanied by altered transcriptional networks and functional impairment [36,106]. Further more, the reduction in aCap/gCap cells in IPF is accompanied by significant downregulation of FOXF1 in residual endothelial cells, leading to inactivation of the FOXF1/R-Ras axis: loss of R-Ras-mediated VE-cadherin stability triggers barrier disruption, promoting aberrant endothelial secretion of profibrotic mediators such as CCL2, IL-6, and TNF-α, which actively drive fibroblast activation and macrophage recruitment [107]. Meanwhile, other researchers isolated pulmonary endothelial cells from bleomycin-induced PF mice and found that injury markers (vWF, PAI-1, MMP-12) and profibrotic mediators (TGF-β, CTGF, PDGF-C) remained elevated throughout the inflammatory and fibrotic phases, while NO and prostacyclin (PGI_2_) production was markedly attenuated. Endothelial cells derived from fibrotic lungs showed aberrant hyperresponsiveness to TGF-β, with increased collagen secretion and upregulated α-SMA, suggesting they are in an EndoMT “primed” state [108]. Consistent with this, Hashimoto et al. identified α-SMA^+^ fibroblast-like cells derived from lung endothelial cells in bleomycin-induced PF lesions, supporting the occurrence of endothelial-to-mesenchymal transition (EndMT) in vivo [109]. In lung tissue from patients with SSc-associated PF, CD31/vWF and α-SMA/Col I co-expressing cells were also detected [110]. Hu et al. discovered that endothelial-specific CD38 knockout activated NAD^+^-SIRT3 signaling, thereby suppressing the TGF-β-Smad3 pathway and attenuating bleomycin-induced EndMT and fibrosis [111]. However, this view has been challenged by fate-mapping studies that directly quantified the origins of myofibroblasts in mouse models of PF. Using Pdgfrb-CreER-mediated lineage tracing, Chandran et al. showed that pre-existing PDGFR-β^+^ mesenchymal cells give rise to the vast majority of α-SMA+ interstitial myofibroblasts after bleomycin injury, whereas cells that already express α-SMA before injury contribute no more than approximately 10% [112]. More recently, Tsukui et al. generated Scube2-CreER mice, which specifically mark alveolar fibroblasts, and found that these cells expanded markedly after bleomycin injury and gave rise to essentially all emergent pathological fibroblast subsets, while endothelial cells, together with adventitial fibroblasts, pericytes, epithelial cells, and hematopoietic cells, were explicitly excluded as major sources [113]. In the same vein, Fang et al. combined Lepr-CreERT2 lineage tracing with single-cell transcriptomics and identified Lepr^+^ fibroblasts, whose major constituent is Scube2^+^ alveolar fibroblasts, as a key source of Cthrc1^+^Postn^+^ pathological fibroblasts in both bleomycin- and silica-induced pulmonary fibrosis. Conditional deletion of Runx2 with Lepr-Cre, Lepr-CreERT2, or Scube2-CreERT2 consistently reduced fibrotic areas, and genetic ablation of Postn+ pathological fibroblasts likewise attenuated fibrosis, confirming the functional dominance of this fibroblast population [114]. Collectively, these studies converge on resident fibroblast lineages as the dominant source of myofibroblasts, a conclusion echoed by a recent synthesis of lineage-tracing data in the single-cell era, which judged the endothelial contribution to pathological myofibroblasts, in terms of cell numbers, to be limited relative to resident mesenchymal lineages [115]. Taken together, EndMT occurs in PF but contributes only moderately to the myofibroblast pool, although even limited endothelial plasticity may aggravate fibrosis by disrupting the alveolar–capillary barrier and paracrine homeostasis.

Endothelial injury can also disrupt the alveolar–capillary barrier, and this shifts regenerative repair toward fibrosis. This barrier is composed of the alveolar epithelium, basement membrane, capillary endothelium, and the junctions between them, and it is essential for maintaining gas exchange, restricting fluid/protein leakage, and controlling inflammatory cell transmigration across the vasculature [71]. PCECs make up a selectively permeable barrier under normal conditions, and they also maintain alveolar microenvironmental homeostasis by producing angiocrine factors [100,116]. When endothelial cells are injured, vascular permeability increases greatly, and fluid rich in protein, coagulation proteases, and inflammatory mediators escapes into alveolar spaces and the interstitium, which breaks barrier integrity, forms a procoagulant microenvironment, and helps fibrin deposition and provisional matrix formation. It has been shown in clinical studies that the bronchoalveolar lavage fluid (BALF) protein/plasma protein ratio (PPI) is markedly raised in IPF patients and is linked to poor prognosis [117]. With persistent injury, these pathological changes progress from increased permeability to structural vascular loss: microvascular endothelial apoptosis increases, lesion vascular density becomes inversely correlated with fibrosis severity, and significant capillary rarefaction occurs. In advanced stages, the pulmonary capillary cross-sectional area within lesions progressively decreases and may disappear entirely, with mature fibrotic foci almost entirely lacking vascular structures, leading to insufficient perfusion in lesion areas and formation of a localized hypoxic microenvironment [118,119].

Under hypoxic conditions, hypoxia-inducible factor-1 alpha (HIF-1α) builds up steadily because hydroxylation and degradation are suppressed, and it moves into the nucleus to form a working heterodimer with HIF-1β. This heterodimer attaches to hypoxia-response elements (HREs) and turns on transcription of downstream target genes. In PF, HIF-1α can directly increase profibrotic factors such as plasminogen activator inhibitor-1 (PAI-1), tissue inhibitor of metalloproteinases-1 (TIMP-1), and connective tissue growth factor (CTGF), and together these factors promote too much ECM deposition and stop the fibrinolytic system [120]. Hypoxia can also directly promote pulmonary fibroblast proliferation and G1/S phase transition through the HIF-2α–NFATc2 pathway [121]. Thus, capillary rarefaction and hypoxia-driven profibrotic signals strengthen one another, forming a vicious “vascular loss–hypoxia–fibrosis” cycle.

### 4.3. Macrophages: A Critical Hub Linking Injury, Inflammation, and Fibrosis

**Figure 3:**
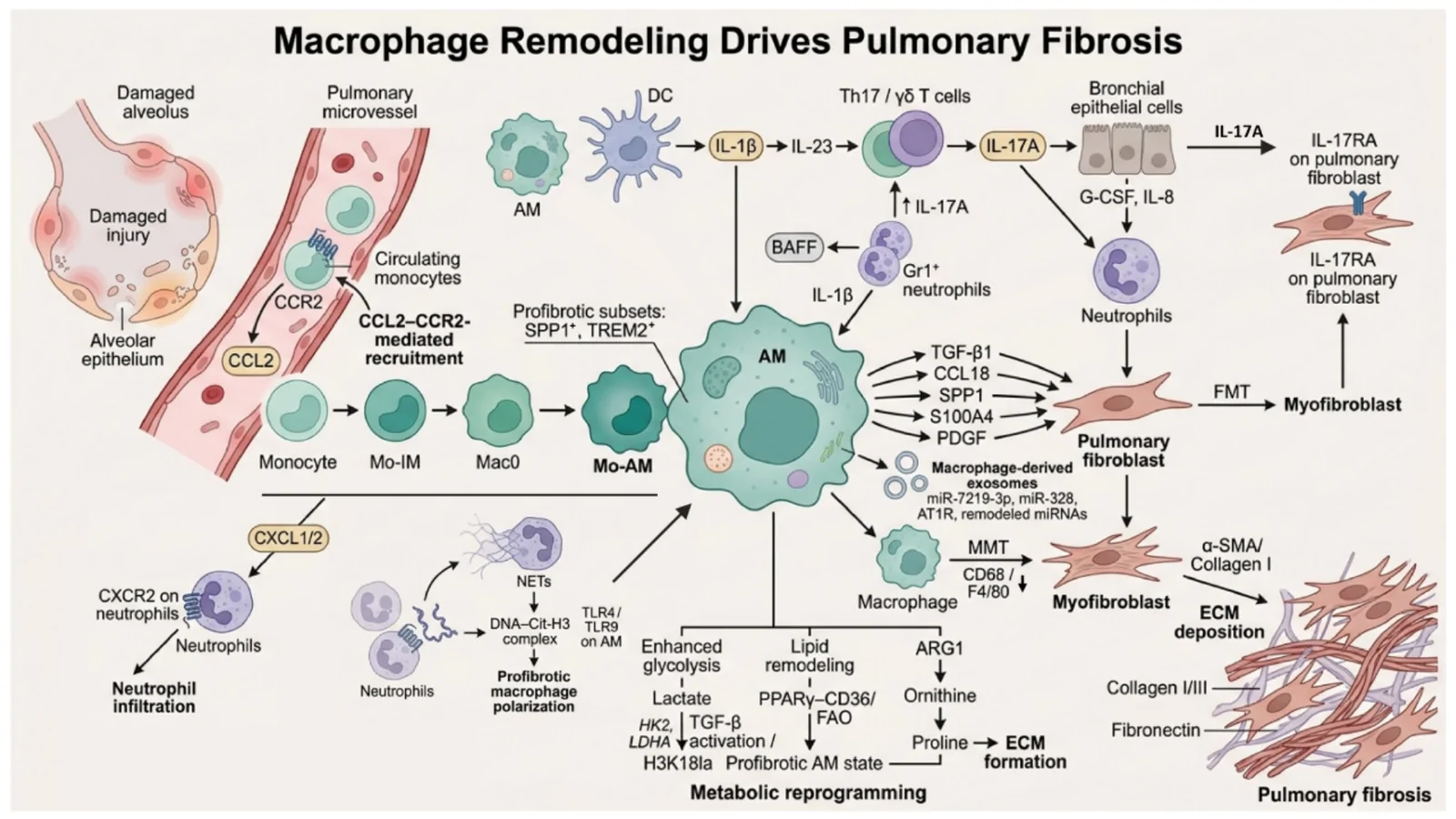
Macrophage remodeling in pulmonary fibrosis. After lung injury, CCL2 attracts circulating monocytes through CCR2 into the lung tissue. These monocytes develop into different macrophage types (Mo-IM and Mac0) and finally become monocyte-derived alveolar macrophages (Mo-AMs). Mo-AMs and dendritic cells activate the IL-1β–IL-23–Th17/γδ T cell–IL-17A pathway. Gr1^+^ neutrophils release BAFF, which increases IL-17A and IL-1β levels. AM-derived CXCL1/2, along with IL-17A-induced G-CSF and IL-8, recruit more neutrophils. Neutrophils release NETs that activate TLR4/TLR9 on AMs and promote profibrotic macrophage changes. These activated macrophages then promote fibroblast activation through secreted factors (TGF-β1, CCL18, SPP1, S100A4, PDGF), exosomes, and metabolic changes, leading to myofibroblast formation, collagen and fibronectin production, and pulmonary fibrosis. (Abbreviations: AM, alveolar macrophage; DC, dendritic cell; Mo-IM, monocyte-derived interstitial macrophage; Mo-AM, monocyte-derived alveolar macrophage; NETs, neutrophil extracellular traps).

PF does not arise simply from autonomous fibroblast expansion; rather, it develops within a complex pathological niche, and this happens against a background of chronic persistent injury, inflammatory imbalance, and aberrant repair [113]. DAMPs can be sensed by macrophages to amplify inflammation, and macrophages can directly influence epithelial repair, endothelial barrier stability, and fibroblast activation through paracrine factors, matrix-remodeling enzymes, exosomes, and metabolic products [122,123]. In this way, macrophages serve as a critical regulatory hub that links alveolar injury, immune imbalance, and the fibrotic repair shift (Figure 3).

#### 4.3.1. Macrophage Heterogeneity Remodeling

Normal lung macrophages mainly comprise tissue-resident alveolar macrophages (AMs) and interstitial macrophages (IMs). AMs originate from embryonic yolk sac or fetal liver monocytes, and they differentiate into long-lived cells postnatally via the granulocyte-macrophage colony-stimulating factor (GM-CSF)/peroxisome proliferator-activated receptor gamma (PPAR-γ) signaling axis. During homeostasis, AMs maintain themselves through local self-proliferation, reside in the alveolar space, and limit excessive inflammation by clearing apoptotic cells, maintaining surfactant homeostasis, and secreting anti-inflammatory mediators. IMs are distributed in the lung interstitium and around blood vessels and airways, and they participate in immune surveillance and tissue homeostasis [124].

During PF progression, persistent epithelial injury and inflammatory stimuli promote the recruitment of circulating monocytes into the lung via C-C chemokine receptor 2 (CCR2)-dependent pathways, where they differentiate into monocyte-derived alveolar macrophages (Mo-AMs) [22,36,125,126,127]. scRNA-seq has identified a class of transitional profibrotic macrophages in IPF and bleomycin models that possess dual characteristics of monocytes and AMs, including highly expressing profibrotic molecules such as SPP1 and MMP9, suggesting the existence of a functional transition intermediate during monocyte-derived cell differentiation in AMs [128]. Recent lineage tracing studies using the CD68-rtTA and Ms4a3-CreER dual recombinase system have tracked the dynamic fates of macrophages from different origins during lung injury repair in vivo, confirming that circulating monocytes entering the lung interstitium do not directly differentiate into AMs but first differentiate into monocyte-derived interstitial macrophages (Mo-IMs), then pass through a transitional macrophage subpopulation named Mac0—this subpopulation highly expresses lysosomal/antigen presentation genes (Ctsb, H2-Aa, Cd74) and ECM interaction genes (Fn1, Cd44, Spp1)—and finally transform into Mo-AMs, completing the spatial migration and identity conversion from interstitium to alveolar space [129]. These infiltrating Mo-AMs can persist in the lung for extended periods and gradually replace resident AMs, promoting fibrosis [22,129].

The replacement of resident AMs by Mo-AMs is only the first step in macrophage landscape remodeling. Driven by the local microenvironment, these cells further differentiate into multiple functionally specialized subpopulations. scRNA-seq has identified at least SPP1^+^, TREM2^+^, and other profibrotic subpopulations [36,37,38,129,130]. Morse et al. identified a macrophage subpopulation characterized by dual high expression of SPP1/MERTK. This population exists at a small proportion within the interstitial macrophage component in normal lung tissue, but in IPF patients, its proportion increases in a gradient manner, reaching the highest level in the lower lobes with more severe fibrosis (approximately 25%) [38]. And its proliferative activity is markedly elevated (from 0.072% in normal lungs to 3.50% in the lower lobes), becoming the predominant proliferative macrophage population in IPF lower lobes [38]. Immunofluorescence shows that the SPP1 protein is abundantly deposited in fibrotic foci and spatially co-localizes with fibroblast foci and α-SMA^+^ myofibroblasts, further supporting the important role of this subpopulation in IPF myofibroblast activation [38]. These findings have been corroborated and extended by spatially resolved transcriptomics. Franzén et al. performed spatially resolved transcriptomic mapping of human and mouse fibrotic lungs and found that profibrotic macrophages are enriched within fibrotic and honeycombing regions, with chemokine signals peaking at the leading edge of fibrosis and suggesting local recruitment of profibrotic macrophages to the fibrotic border [90]. Mayr et al. resolved the pathologic niches of IPF lungs, identifying SPP1^+^ airway macrophage niches, localized within fibrotic distal airways and adjacent to the fibrotic niche, as a recurrent feature of IPF tissue [131]. Moreover, in a spatial atlas of 35 lungs (control and fibrotic) comprising 1.6 million cells, Vannan et al. showed that changes in macrophage polarization track the distal lung remodeling severity gradient [132]. The profibrotic activity of SPP1^+^ macrophages is further underpinned by a defined ligand-receptor mechanism with multiple experimentally testable nodes. Upstream, injured AT2 cells secrete sonic hedgehog (SHH), which induces SPP1 transcription in macrophages through GLI family transcription factors; SHH silencing reduces macrophage SPP1 production and protects mice from fibrosis, while SHH neutralization suppresses macrophage alternative activation, identifying an epithelial-macrophage feed-forward loop that initiates SPP1^+^ macrophage accumulation [133]. Downstream, macrophage-derived osteopontin acts on alveolar epithelial cells via CD44, triggering FAK/AKT signaling and snail1-mediated profibrotic protein expression, and OPN knockdown in the bleomycin model, or FAK inhibition in precision-cut lung slices, attenuates fibrogenic protein expression and experimental fibrosis [134]. Consistently, OPN stimulates collagen I expression in lung fibroblasts [135], and SPP1 overexpression in macrophages is sufficient to drive fibroblasts toward an activated, ECM/CAF-like phenotype in co-culture [136]. Yang et al. demonstrated that SPP1 promotes macrophage M2 polarization via the JAK2/STAT3 pathway [137]. The knockdown of SPP1 suppresses the expression of IL-10, TGF-β and CD206, and its conditioned medium can cause EMT in BEAS-2B and WI-38 cells, which is characterized by the downregulation of E-cadherin and upregulation of N-cadherin, Vimentin, and COL3A1. Both SPP1 knockdown and the STAT3 inhibitor (stattic) can revert this effect [137]. In a mouse model of pulmonary fibrosis induced by bleomycin, osteopontin(OPN)-deficient mice exhibited significantly reduced lung inflammation and fibrosis with reduced type I collagen expression, genetically validating the central driving force of SPP1/OPN in PF progression [135,138]. Moreover, OPN released by CD163^+^ macrophages is able to stimulate the expression of collagen I in lung fibroblasts, and the inhibition of OPN inhibits this process, indicating that OPN is an important mediator of ECM remodeling [135]. Clinically, OPN levels are elevated in IPF lungs and correlate with disease severity and poor prognosis [134]. This SPP1^+^ profibrotic program is also conserved across etiologies, as post-COVID-19 lungs harbor CD163^+^ monocyte-derived macrophages with a profibrotic transcriptional phenotype closely resembling that in IPF [139]. Beyond the lung, SPP1^+^ macrophages define a matrisome-associated polarization state shared by multiple fibrotic organs [140], and in models of cardiac and renal fibrosis, platelet-derived CXCL4 drives the differentiation of SPP1^+^ macrophages that activate myofibroblasts through Spp1, Fn1, and Sema3 signaling networks [141]. Whether these mechanisms operate in pulmonary fibrosis remains to be determined.

Significant advances have been made in the understanding of the mechanism of TREM2^+^ Mo-AMs in PF. TREM2 is mainly expressed on Mo-AMs, and is highly upregulated in patients with IPF and bleomycin models. TREM2-high-expression macrophages are characterized by high levels of scar-associated macrophage markers and anti-apoptotic mediators. Mechanistic experiments show that TREM2 sustains the survival of Mo-AMs through the activation of the AKT/ERK/mTOR signaling pathway, stimulates the secretion of profibrotic factors like TGF-β1 and SPP1, and suppresses alveolar epithelial regeneration [130]. It was also shown that TREM2 controls the metabolism of sphingolipids in lung macrophages and expression of chemokines like CCL2 to facilitate inflammatory cell recruitment and progression of fibrosis, and TREM2 gene knockout or soluble TREM2 administration can suppress bleomycin-induced PF [142]. Causal evidence at the cellular level has been strengthened considerably. Cui et al. showed that surfactant-derived sphingomyelin engages TREM2 to sustain Mo-AM survival. Using both global and Mo-AM-specific TREM2 knockout mice, they further showed that loss of TREM2 in Mo-AMs protects against bleomycin-induced fibrosis [130]. In addition, administration of soluble TREM2, which acts as a decoy receptor to block surfactant lipid engagement and induce Mo-AM apoptosis, or administration of a TREM2-blocking antibody attenuated fibrosis even when given after bleomycin injury [130]. These results support the necessity of TREM2 for Mo-AM survival and the therapeutic potential of targeting this axis. In parallel, TREM2 silencing blocked STAT6-dependent M2 polarization and reduced fibrogenic outputs (TGF-β, PDGF, Col I, α-SMA) in vivo, and high TREM2 expression in IPF patients predicted worse survival [143]. However, TREM2 function may be context-dependent. TREM2^+^ lipid-associated macrophages have been reported to promote fibrosis resolution in liver fibrosis [144], suggesting that the profibrotic role of TREM2^+^ Mo-AMs may be lung-specific and should not be generalized across organs. Compared with SPP1^+^ macrophages, for which macrophage-specific causal evidence is limited, the TREM2^+^ Mo-AM subset itself was causally validated by Mo-AM-specific conditional deletion [130]. However, these findings still need to be validated in multiple models of pulmonary fibrosis.

#### 4.3.2. Macrophage-Driven Fibrosis

##### Activation of Fibroblasts by Paracrine

The direct activation of fibroblasts by macrophages via soluble mediators is a central regulatory pathway for the induction and progression of fibrosis. Beyond the well-known TGF-β1 pathway, several new paracrine pathways have been uncovered recently.

TGF-β1 is still the accepted major mediator of fibrosis. In PF progression, macrophages and other immune cells are activated and recruited to the site of lung tissue injury, which constantly release profibrotic mediators like TGF-β1 [19,145]. TGF-β1 stimulates the process of fibroblast-to-myofibroblast transition through activation of the TGF-β1/Smad2/3 signaling pathway in fibroblasts, leading to the expression of α-SMA and the production and deposition of ECM proteins, including collagen and fibronectin, which ultimately leads to pulmonary tissue fibrosis.

Another significant paracrine pathway is the chemokine CCL18 [19,145]. The production of CCL18 is mostly undertaken by AMs of patients with IPF [146,147,148], and it can directly activate fibroblast collagen production via the ERK-Smad3-Sp1 transduction axis [148,149,150]. The newly synthesized type I collagen monomers, in turn, may cause Akt phosphorylation through CD204 (scavenger receptor A) on the surface of macrophages to drive alveolar macrophage polarization to an alternatively activated phenotype and increase the secretion of CCL18 [151]. Clinically, CCL18 is found to be highly expressed in serum and bronchoalveolar lavage fluid (BALF) of IPF patients [152], and Prasse et al. also found serum CCL18 to be the first biomarker that significantly predicts mortality in IPF [153].

S100A4 (or FSP-1) is a significant profibrotic mediator released by alternatively activated AMs that are capable of directly interacting with lung fibroblasts, stimulating their proliferation and myofibroblast conversion [154,155]. This effect can be effectively prevented by pharmacological inhibition using niclosamide and other related agents [155], which suggests that this molecule could be a possible target of PF intervention.

Mia et al. used myeloid-specific gene-editing models to establish that macrophage YAP/TAZ activation can directly transactivate the MBD2 promoter to drive TGF-β1-pSMAD2 signaling [156]. Experiments using the conditioned medium validated the hypothesis that MBD2 deficiency greatly suppresses the capacity of macrophages to cause fibroblast-to-myofibroblast transformation, as measured by decreased numbers of cells expressing α-SMA and type I collagen [156]. Furthermore, knockdown of Mbd2 inhibits pSMAD2 activation in fibroblasts, and TGF-β1-neutralizing antibodies inhibit the formation of myofibroblasts and collagen synthesis [156]. Besides this, CCL2 blockade also prevents YAP-mediated macrophage recruitment and alleviates fibrosis [156].

##### Remote Regulation via Exosomes

Exosomes and extracellular vesicles (EVs) are nanocarriers for intercellular communication, transporting proteins, lipids and nucleic acids (particularly miRNA) between cells to mediate non-contact remote regulation. In a silicosis model, macrophage exosomes contain miR-7219-3p, which induces fibroblast-to-myofibroblast transdifferentiation by targeting SPRY1, thus activating the ERK/MAPK pathway [157]. Another study identified that M2 macrophage-derived exosomes contain miR-328, which enhances lung fibroblast proliferation and collagen deposition through downregulation of its target gene FAM13A, thus aggravating PF [158]. Moreover, in a silicosis model, exposure to SiO_2_ nanoparticles can trigger pyroptosis in interstitial macrophages, resulting in changes to their exosomal miRNA profiles. These remodeled miRNA-containing exosomes promote fibroblast-to-myofibroblast transdifferentiation via TGF-β signaling [159]. Macrophage exosomes can also transfer the angiotensin II type 1 receptor (AT1R) to fibroblasts, leading to Ang II production and activation of the local renin–angiotensin system (RAS), contributing to fibrosis [160].

##### Macrophage-to-Myofibroblast Transdifferentiation (MMT)

According to traditional theory, resident fibroblasts are the primary sources of myofibroblasts in PF. Accumulating evidence suggests that macrophages may acquire a myofibroblast-like phenotype through a process termed macrophage-to-myofibroblast transition (MMT). However, this concept requires cautious interpretation, as the strength of the supporting evidence varies considerably across organs. In the unilateral ureteral obstruction (UUO) model, myofibroblasts derived from the myeloid lineage macrophage have been reported to account for 75–80% of the total α-SMA^+^ cell population, of which approximately 65% retain F4/80 expression and produce type I collagen [161]. Similar findings have been reported in cardiac [162], liver [163], and pulmonary fibrosis [164], suggesting that MMT may be a conserved process in organ fibrosis, although direct lineage-tracing confirmation outside the kidney remains limited. The defining feature of MMT cells is the co-expression of macrophage lineage markers (CD68, F4/80) and myofibroblast functional markers (α-SMA). However, marker co-expression alone cannot distinguish genuine transdifferentiation from macrophages that have phagocytosed α-SMA^+^ cellular debris, and rigorous fate-mapping evidence for MMT in PF is still scarce. In line with this, single-cell RNA sequencing of fibrotic lungs has identified a transitional, profibrotic macrophage subset that expresses a gene profile intermediate between monocyte-derived and alveolar macrophages and localizes to the fibrotic niche; however, these cells retain macrophage identity and promote fibrosis through paracrine PDGF-AA signaling rather than converting into myofibroblasts [128].

Tang et al. identified a candidate molecular mechanism potentially underlying MMT in IPF [164]. Following activation of the receptor tyrosine kinase MERTK on macrophages by its ligand GAS6, an SPP1-SRC-TKS5 signaling cascade is initiated: SPP1 recruits and activates SRC, which, in turn, phosphorylates TKS5 to promote podosome assembly and cytoskeletal remodeling, ultimately driving MMT. Macrophage-specific knockout of Mertk or AAV9-mediated knockdown of Tks5 effectively suppresses MMT and PF progression both in vitro and in vivo [164]. Nevertheless, these interventions attenuate fibrosis without directly quantifying, through fate mapping, the macrophage-derived fraction of the α-SMA^+^ myofibroblast pool; they therefore support, but do not conclusively demonstrate, a direct macrophage-to-myofibroblast lineage transition. Macrophage-specific inducible lineage-tracing studies are thus needed to establish MMT as a bona fide source of myofibroblasts in PF.

#### 4.3.3. Macrophage–Neutrophil Interactions

Macrophages and neutrophils constitute the most active innate immune dialog axis in the pulmonary fibrosis microenvironment. Through chemokine-NET-cytokine signaling, they form a self-amplifying positive feedback loop that serves as the core driving force for the transition from inflammation to fibrosis.

##### Macrophage-Mediated Recruitment and Activation of Neutrophils

In fibrotic lungs, macrophages are key upstream regulators of sustained neutrophil infiltration [165]. Warheit et al. found that tissue-resident macrophages significantly upregulated CXCL2 in BLM-induced fibrotic lungs, driving neutrophil infiltration via CXCR2 [166]. Macrophage-derived CXCL1 is also a key chemokine for neutrophil recruitment in the lungs [167]. In addition, following ablation of nociceptor sensory neurons, AMs produce vasoactive intestinal peptide (VIP), which induces TGF-β1 and expands the profibrotic Siglec-F^+^ neutrophil subset [168]. In a silica model, NLRP3 inflammasome activation in AMs releases interleukin-1β (IL-1β) and interleukin-18 (IL-18), driving neutrophil airway infiltration and neutrophil elastase (NE) activation, thereby mediating myofibroblast activation via TGF-β [169].

##### Neutrophil Extracellular Traps (NETs) and Reverse Regulation of Macrophages

NETs are extracellular web-like structures secreted by activated neutrophils, in which decondensed chromatin DNA is used as a scaffold and granular proteins such as myeloperoxidase (MPO), NE, and citrullinated histone H3 are embedded [170]. Pathological conditions can lead to impaired clearance or continued formation of NETs which promote sterile inflammation and fibrosis [170,171]. The DNA-Cit-H3 complexes in the NETs bind to Toll-like receptor 4 (TLR4) and Toll-like receptor 9 (TLR9) on the surface of macrophages, stimulating NF-κB and causing secretion of profibrotic molecules like IL-6, IL-1β and TGF-β, thus promoting macrophage polarization towards a profibrotic phenotype [170,172]. NETs may also induce macrophage polarization to an M2-like profibrotic phenotype in a MerTK-dependent fashion in a model of systemic sclerosis [173]. Dense ECM can also cross-link with NETs to create NET-ECM complexes that hinder inflammatory resolution and give long-term activation signals to macrophages [174]. Riehl et al. discovered that externalized histones stimulate PF through a platelet-macrophage loop of TGF-β1 and IL-27 [175]. Increased glycolysis facilitated by glucose transporter 1 (GLUT1) may contribute to the formation of NETs, thus impairing phenotypic switching of macrophages and enhancing profibrotic interactions between macrophages and fibroblasts [176].

NETs can also directly target fibroblasts: Shao et al. discovered that NETs directly induce myofibroblast differentiation and collagen release via the TLR9/NF-κB/IL-6 axis [177]. The DAMP HMGB1 associated with the NET can also directly promote lung fibroblast-to-myofibroblast transition through NF-κB-mediated TGF-β1 release [178]. The NE inhibitor, sivelestat, can suppress BLM-induced PF by blocking TGF-β activation and inflammatory cell recruitment [179].

##### IL-17A-Mediated Neutrophil–Macrophage–T Cell Triad Interaction Network

Interleukin-17A (IL-17A) is an important inflammatory node linking T cells, neutrophils, and macrophages, and is produced mainly by T helper 17 (Th17) cells and gamma-delta (γδ) T cells [19,180]. AMs and dendritic cells (DCs) produce IL-1β early after lung injury, inducing interleukin-23 (IL-23) to drive γδ T cells and Th17 cells to produce IL-17A [180]. In this microenvironment, TGF-β can also drive naive CD4^+^ T cell differentiation toward the Th17 phenotype by suppressing SOCS3 expression and enhancing and prolonging STAT3 phosphorylation, thereby upregulating RORγt [181]. IL-17A acts on stromal cells such as bronchial epithelial cells and lung fibroblasts, inducing expression of CXCL1, CXCL2, G-CSF, and IL-8, thereby recruiting neutrophils into lung tissue and participating in the progression of pulmonary fibrosis [182,183]. Furthermore, in human cells, IL-17A directly binds to interleukin-17 receptor A (IL-17RA) on lung fibroblasts and promotes their proliferation, ECM synthesis, and myofibroblast transdifferentiation via NF-κB/JAK2 signaling. Murine studies further demonstrate that IL-17A can selectively induce the Thy-1^−^ subset to differentiate into myofibroblasts by downregulating Thy-1 [184,185]. IL-1β and IL-17A synergistically induce infiltrating Gr1^+^ neutrophils to release the B cell activating factor (BAFF), which, in turn, dose-dependently amplifies T cell IL-17A responses and forms a reciprocal amplification loop with IL-1β, jointly promoting TGF-β1 release and collagen deposition, constituting a self-amplifying inflammation–fibrosis loop centered on neutrophil–IL-17A^+^ T cell–macrophage/DCs [186]. Wilson et al. confirmed that BLM- and IL-1β-induced pulmonary fibrosis is strictly dependent on IL-17A, and its deficiency significantly attenuates neutrophil infiltration and collagen deposition [182]. Mi et al. further revealed that targeted blockade of IL-17A promotes fibrosis resolution through both TGF-β1-dependent and -independent mechanisms (such as restoring autophagy to promote collagen degradation) [187].

#### 4.3.4. Macrophage Metabolic Reprogramming

The key process through which macrophages sustain a profibrotic phenotype is metabolic reprogramming. Macrophages in the fibrotic microenvironment reprogram glucose, lipid and amino acid metabolic circuits, transforming metabolic intermediates into signals that stimulate fibroblasts through paracrine mechanisms. The profound interconnection between metabolic reprogramming and epigenetic changes can also entrench the profibrotic phenotype, creating a self-perpetuating pathological loop [188,189,190].

##### The Glycolysis–Lactate Axis and Fibrosis

Both IPF patient alveolar macrophages and BLM-induced mouse models have increased aerobic glycolysis [188,191,192,193]. THBS1 mRNA is stabilized by an N6-methyladenosine (m6A)-dependent mechanism that results in high levels of THBS1 protein, which then activates key glycolytic enzymes such as HK2, LDHA, and PKM2 through interaction with THBS1-TLR4 to promote macrophage polarization towards M2-like phenotype and increase lactate production [194]. Glycolysis is enhanced to supply ATP and biosynthetic precursors to activate macrophages and produce profibrotic mediators (including TGF-β1 and MMPs) [195,196]. Lactate, a significant glycolytic metabolite, can indirectly stimulate latent TGF-β by decreasing extracellular pH, thus causing fibroblast-to-myofibroblast transition (FMT) [197]. Moreover, histone lactylation in macrophages may induce secretion of profibrotic proteins like VEGFA, leading to EMT in PM2.5 exposure models [198].

Concurrently, activated myofibroblasts enhance aerobic glycolysis through metabolic reprogramming, producing and secreting large amounts of lactate, thereby generating a localized high-lactate concentration in the fibrotic microenvironment. After these exogenous lactate molecules enter macrophages via monocarboxylate transporters (MCTs), they serve as epigenetic regulatory signals and induce histone H3 lysine 18 lactylation (H3K18la) under the catalysis of the histone acetyltransferase p300. Chromatin immunoprecipitation analysis shows that H3K18la is specifically enriched in the promoter regions of profibrotic genes such as Arg1, activating their transcriptional expression through chromatin remodeling. Moreover, p300 knockdown significantly attenuates lactate-induced histone lactylation and downstream profibrotic gene expression [199,200]. Thus, myofibroblast-derived lactate drives macrophage profibrotic activation through epigenetic reprogramming and, together with profibrotic factors secreted by macrophages, constitutes a positive feedback regulatory loop of “myofibroblast–lactate–macrophage” [199,201]. LDHA inhibition by GNE-140 markedly improves lung collagen deposition and fibrotic severity in a PM2. 5-induced pulmonary fibrosis mouse model, indicating that targeting this axis may have antifibrotic effects [198]. Lactate has the ability to directly alter the major glycolytic enzyme PKM2 as well. It was found that lysine 62 lactylation of PKM2 stabilizes its tetrameric form, increases the activity of pyruvate kinase, and at the same time, decreases its nuclear transport [202]. This non-histone lactylation reaction inhibits the Warburg effect in macrophages and synergistically drives their shift from the pro-inflammatory M1 phenotype to the profibrotic M2 phenotype [202,203].

##### Lipid Metabolism Remodeling and Fibrosis

In homeostasis, AMs sustain a PPARγ-FABP4 transcriptional program under the influence of GM-CSF signaling and effectively internalize and metabolize surfactant lipids to avoid lipid droplet accumulation. In pulmonary fibrosis, this lipid processing program is disrupted, and AMs undergo lipid droplet accumulation (foam cell formation), oxidized phospholipid accumulation, and cholesterol efflux dysfunction, transitioning to a profibrotic phenotype [204,205]. Apoptotic cells in this process trigger PPARγ activation and expression of target molecules like CD36 [206], which facilitate macrophage uptake of fatty acids and M2-like polarization. On the other hand, CD36 deficiency suppresses the M2 program (less Arginase-1), suppresses the production of Th2 cytokines such as IL-4, IL-9 and IL-13, and suppresses lung fibrosis [207]. After M2 polarization, macrophages stimulate lung fibroblasts through paracrine release of mediators like TGF-β1 and PDGF, which promote FMT and deposition of type I and III collagen. There is a great cell-type specificity of the profibrotic effects of dysregulation of lipid metabolism [19,145]. At the whole-tissue level in IPF lungs, metabolomic analyses reveal elevated free fatty acid levels and impaired carnitine shuttle function, suggesting compromised mitochondrial β-oxidation capacity [208,209]. Nevertheless, certain profibrotic macrophage subsets (including M2-like alternatively activated macrophages) exhibit increased fatty acid oxidation (FAO), which fits into the classical paradigm that alternatively activated macrophages depend on FAO and mitochondrial oxidative phosphorylation to sustain their phenotype [210]. S100A4 is primarily expressed and secreted by alternatively activated AMs in pulmonary fibrosis [155], and S100A4 has been demonstrated to upregulate CD36 and CPT1 through a PPARγ-dependent mechanism, thus promoting fatty acid uptake and oxidation [211], potentially supplying metabolic support to maintain secretion of profibrotic mediators like TGF-β. In addition, TGF-β1 induces the expression of FASN via a Smad2/3-independent mTORC1 pathway. FASN is the major rate-limiting enzyme in de novo lipogenesis; high levels of its expression may provide activated fibroblasts with lipid substrates needed for their proliferation [212]. Conversely, metabolomic analysis has revealed that the levels of stearic acid are very low in the lung tissue of IPF patients. Exogenous stearic acid is able to block TGF-β1-mediated α-SMA and collagen I expression through downregulating Smad2/3 phosphorylation and attenuating pulmonary fibrosis in the BLM model [213]. These results imply that the synergistic effect of TGF-β1/mTORC1-FASN axis-mediated profibrotic lipid synthesis and endogenous antifibrotic lipid depletion could be the metabolic foundation underlying excessive ECM deposition.

##### Amino Acid Metabolism and Collagen Synthesis

Multiple amino acid metabolites are elevated in IPF patient lung tissue and exhaled breath, correlating with disease severity. Metabolomic analysis reveals that proline levels are significantly increased in IPF lung tissue, accompanied by upregulated ornithine aminotransferase (OAT) expression and an increased proline/ornithine ratio; OAT expression negatively correlates with the degree of lung function deterioration (FVC%) [214]. Molecular breath analysis further confirms elevated proline and hydroxyproline levels in exhaled breath [215]. In addition, glutamate is significantly elevated in IPF lung tissue and glutamine shows an increasing trend, whereas arginine itself does not change markedly. However, its downstream metabolites including 4-hydroxyproline, putrescine, spermidine, and creatine, are significantly increased [123,209,214,215]. These alterations in amino acid metabolism constitute an important feature of metabolic reprogramming in IPF.

In pulmonary fibrosis, glutamine is converted to glutamate by GLS1, which subsequently generates α-ketoglutarate and proline, providing direct precursors for collagen synthesis [216]. SNX9-mediated endocytosis of CTSK activates SMAD3 and upregulates GLS1 expression, thereby enhancing glutamine flux toward proline and promoting collagen synthesis in fibroblasts [217]. Thus, GLS1 inhibitors can effectively attenuate this pathway and reduce collagen deposition in the BLM model [216,218]. The arginine–proline metabolic axis is upregulated in lung tissue (with elevated enzymes including OAT, PYCR1, and arginase), whereas arginine itself is not significantly upregulated [209], suggesting that arginine may be rapidly shunted into the proline synthesis pathway. ARG1 expression is significantly upregulated in IPF patient lung tissue [219,220]. Myeloid cells convert arginine to ornithine and urea via ARG1, and ornithine can be taken up by fibroblasts, converted to pyrroline-5-carboxylate (P5C) by OAT, and subsequently reduced to proline by P5C reductases (including PYCR1) [220,221]. Proline and 4-hydroxyproline are crucial for triple helix stabilization. The prolyl-4-hydroxylase (P4H) catalyzed 4-hydroxylation of proline stabilizes the triple helix structure through stereoelectronic effects and is essential for thermal stability of collagen [222,223]. Lung fibroblasts can also activate de novo proline synthesis via glutamine metabolic reprogramming to provide substrates for collagen production [216]. At the same time, fibroblasts can also uptake myeloid cell-derived ornithine for proline synthesis, driving collagen deposition and fibrosis through immune–mesenchymal metabolic interactions [220].

##### Fibrotic Signals Are Enhanced by Metabolic–Epigenetic Coupling

The mechanism that sustains the macrophage profibrotic phenotype is the coupling of epitranscriptomic modifications with metabolic reprogramming. This network of coupling defines the direction of polarization in macrophages. IGF2BP1, an m6A reader, identifies and stabilizes THBS1 mRNA to promote glycolytic reprogramming and M2-like polarization through the THBS1-TLR4 axis [194]. On the other hand, METTL3-mediated m6A modification of STAT1 facilitates M1-type pro-inflammatory activation [224], which implies that the same modification machinery can regulate the dynamic balance between pro-inflammatory and profibrotic programs via different substrates. Moreover, CPT1A demethylation by ALKBH5 can promote fatty acid oxidation and aid in M2-like polarization, a process that has been described in colorectal cancer [225], indicating possible conservation in the fibrotic microenvironment.

Other cell types also rely on this network to drive fibrosis. In lung fibroblasts, METTL3 promotes KCNH6 mRNA translation via YTHDF1 to drive FMT [226]. The synergistic action of H3K18 lactylation and m6A can also promote arsenite-related pulmonary fibrosis, suggesting cross-layer dialog between epigenetic hierarchies [227]. In alveolar epithelial cells, 1-nitropyrene-induced mitochondrial ROS overproduction triggers ALKBH5 SUMOylation and degradation, leading to elevated m6A modification and enhanced translation of FBXW7, which subsequently mediates TRF2 ubiquitination and degradation, triggering telomere damage and accelerated cellular senescence [228]. This cascade can be effectively blocked by the mitochondrial-targeted antioxidant Mito-TEMPO.

### 4.4. Fibroblast Activation

#### 4.4.1. Fibroblast Heterogeneity: From a “Single Population” to a “Multi-Subtype Atlas”

Lung fibroblasts exhibit significant heterogeneity. Travaglini et al. performed high-throughput scRNA-seq on healthy human lungs and systematically identified five transcriptionally distinct fibroblast/stromal cell subpopulations within the mesenchymal compartment [229]. Alveolar fibroblasts (SPINT2^+^/FGFR4^+^/GPC3^+^) localized to alveolar interstitium, which are excitable and participate in local immune regulation. Adventitial fibroblasts (PI16^+^/SFRP2^+^/SERPINF1^+^) that are found around the vascular adventitia and proximal airways are enriched in morphogen and growth factor signaling. Lipofibroblasts (PLIN2^+^/APOE^+^) are scattered throughout the alveolar areas, defined by lipid storage and metabolism. Myofibroblasts (ACTA2^+^/ASPN^+^/WIF1^+^) are localized to alveolar ducts, which predominate in ECM synthesis, and maintenance of the alveolar structure; fibromyocytes (MYH11^+^/CNN1^+^/TAGLN^+^) are localized to the proximal airway wall and intermingled with smooth muscle, which mainly mediate contraction. These subpopulations show considerable variations in anatomical localization, selective gene expression and functional features, all of which form a spatially and functionally specialized heterogeneity atlas of lung stromal cells that sustains the homeostasis of ECM [229].

The “multi-subtype balance” of lung fibroblasts is altered in fibrotic diseases like IPF, which is characterized by a three-dimensional process that involves changes in the proportion of normal subpopulations, appearance of pathological subpopulations and their spatial relocalization. In IPF lungs, the percentage of homeostatic alveolar fibroblasts is dramatically reduced, while COL1A1^+^-, ACTA2^+^- and TAGLN^+^-activated fibroblasts are significantly increased compared to healthy lungs [20,36,37]. This process is termed the fibroblast-to-myofibroblast transition (FMT), during which quiescent fibroblasts express α-SMA (ACTA2), gain a contractile function, and produce abundant collagen and other ECM proteins in response to cytokines like TGF-β, a key event in the development and progression of fibrosis [19]. However, the traditional framework using ACTA2 (α-SMA) as a marker for pathological collagen-producing cells has clear limitations. Through construction of a collagen-producing cell atlas, Tsukui et al. found that ACTA2 and COL1A1 expression were poorly correlated, and smooth muscle cells and pericytes with high α-SMA expression actually showed low collagen expression levels [39]. This paradox suggests that the cells truly driving excessive ECM deposition in fibrotic lungs may be pathological subpopulations that have not yet been fully recognized.

Based on the above re-examination of traditional FMT markers, studies have progressively identified pathological subpopulations specifically emerging in fibrotic lungs. Peyser et al. defined the transcriptional signature of “activated fibroblasts” through scRNA-seq, which is characterized by high expression of POSTN, CTHRC1, and matrix metalloproteinases [230]. In the collagen-producing cell atlas, Cthrc1^+^ fibroblasts were specifically enriched after bleomycin injury, expressed the highest levels of type I collagen and other ECM genes, and localized within fibroblastic foci [39]. This pathological fibroblast population is also detected in IPF and scleroderma patient lungs, and is recognized as a core effector cell driving fibrosis [39].

Currently, there are multiple hypotheses regarding the cellular origins of pathological subpopulations. Tsukui et al. demonstrated that Scube2-CreER-labeled alveolar fibroblasts differentiate into Cthrc1^+^CD9^+^ fibrotic fibroblasts after bleomycin injury, a process conserved in the silicosis model [113]. Fang et al. found that leptin receptor-positive (LEPR^+^) alveolar fibroblasts (including the SCUBE2^+^ subpopulation) are one of the major sources of CTHRC1^+^POSTN^+^ pathological fibroblasts, and the transcription factor RUNX2 participates in this differentiation process; conditional deletion of Runx2 reduces pathological fibroblast generation and attenuates fibrosis [114]. In addition, a high Saa3, Lcn2, Cxcl12 and Ccl2 inflammatory subpopulation of fibroblasts was found in bleomycin-treated mouse and IPF human lungs. Pseudotime analysis suggests that this population is an intermediate state between alveolar fibroblasts and fibrotic fibroblasts (Cthrc1^+^/Col1a1^+^) [113]. Zepp et al. performed single-cell sequencing combined with Axin2/Pdgfrα/Wnt2 lineage tracing, and the results showed that adult lung mesenchymal cells are highly spatially and functionally diverse: Axin2^+^Pdgfrα^−^ mesenchymal cells located around airways and blood vessels (Axin2^+^ myofibrogenic progenitors, AMPs) can massively differentiate into α-SMA^+^ pathological myofibroblasts after bleomycin injury (contributing nearly half), whereas Axin2^+^Pdgfrα^+^ mesenchymal cells located in the alveolar region (mesenchymal alveolar niche cells, MANCs) primarily support AT2 epithelial regeneration [231]. These findings indicate that fibroblasts from different anatomical sites can contribute to fibrosis.

Alongside changes in cellular composition, there is also extensive remodeling of the spatial distribution of fibroblast subpopulations. Notably, the emergence of CTHRC1^+^ pathological fibroblasts is not restricted to IPF. Proliferating CTHRC1^+^ myofibroblasts have also been identified in the fibrotic lungs of systemic sclerosis patients [232]. In post-COVID-19 lung fibrosis, CTHRC1^+^ cells are essentially absent from normal lung but progressively accumulate during the proliferative phase of diffuse alveolar damage, initially within the alveolar septa and subsequently in septal and periductal fibrotic areas, suggesting that they contribute to the rapidly evolving fibrosis in this context [233]. Whether CTHRC1^+^ fibroblasts constitute a shared pathogenic node in other pulmonary fibrotic contexts, such as radiation-induced pulmonary fibrosis and hypersensitivity pneumonitis, warrants further investigation. Comparable CTHRC1^+^ pathological fibroblast subsets have been reported in cardiac and hepatic fibrosis [234,235], indicating that this fibrogenic program is conserved across organs. scRNA-seq experiments have repeatedly revealed CTHRC1^+^ pathological fibroblasts as one of the most enriched profibrotic subpopulations with elevated ECM biosynthetic genes in IPF. Integrative spatial transcriptomics validates that these cells are specifically enriched in fibroblastic foci and dense fibrotic areas and exhibit increased intercellular communication activity [20,36,37,39,89,230]. Likewise, Watanabe et al. identified a WNT5A^+^CTHRC1^+^ myofibroblast subset that is enriched in fibroblastic foci and dense fibrotic areas and is regulated by p21-activated kinase 2 (PAK2). Inhibition of PAK (FRAX486/FRAX597) blocks TGF-β-mediated myofibroblast differentiation and ECM production, indicating that this pathway may be an attractive target for antifibrotic therapy [89]. Tsukui et al. identified that CTHRC1^+^ fibrotic fibroblasts are predominantly located within the alveolar lumen, closely associated with new collagen deposition, and distinct from Pi16^+^ peribronchial/adventitial fibroblasts. These findings were confirmed by RNAscope in situ hybridization analysis of human IPF tissues, which showed that CTHRC1^+^ fibrotic fibroblasts are preferentially localized to fibroblastic foci, while inflammatory fibroblasts (including IL-1/TNF-responsive and IFN-responsive types) are mainly found in foci-interstitium interface [113]. Such findings indicate that different functional fibroblast subsets display highly segregated spatial distributions in the pulmonary fibrosis microenvironment.

Beyond localization, the causal contribution of CTHRC1^+^ fibroblasts to fibrogenesis has been tested by selective depletion. Consistent with their dominant ECM output described above [39], selective depletion of this population was observed, approximately 50% of CTHRC1^+^ fibroblasts were eliminated via Cthrc1-CreER; Rosa26-DTA markedly reduced lung hydroxyproline content and collagen I deposition, formally demonstrating that these cells drive de novo fibrogenesis rather than merely marking fibrotic tissue [113]. At the cellular level, CTHRC1 is a functional driver of the fibrotic fibroblast phenotype. TGF-β1 induces CTHRC1 release from patient-derived fibrotic lung fibroblasts, and recombinant CTHRC1 alone is sufficient to stimulate collagen gel contraction, chemotaxis, and α-SMA/fibronectin expression, whereas CTHRC1 knockdown or pirfenidone treatment prevents these responses [236]. Together with the upstream regulators already discussed, these genetic and cellular findings identify the CTHRC1^+^ fibroblast population and its regulatory circuitry as a tractable therapeutic target. Clinically, circulating CTHRC1 is elevated in patients with IPF and is associated with lower baseline lung function, faster FVC decline, and worse transplant-free survival in two large independent cohorts, supporting its utility as a non-invasive biomarker of pathological fibroblast burden [237]. Interestingly, global Cthrc1 knockout exacerbated, rather than ameliorated, bleomycin-induced pulmonary fibrosis [238]. This discrepancy may reflect differences between whole-body gene deletion and selective cell ablation, as well as context-dependent functions of CTHRC1. How these findings should inform therapeutic targeting of this population remains to be clarified.

Beyond their fixed identities, fibroblast subpopulations are highly phenotypically plastic in the course of fibrosis. El Agha et al. utilized bidirectional lineage tracing of lipofibroblasts (AdrpCre-ERT2) and myofibroblasts (Acta2-Cre-ERT2) and showed that there is a bidirectional conversion between lipofibroblasts and myofibroblasts during the formation of fibrosis. TGF-β1 signaling induces conversion to myofibroblasts, which facilitates the development of fibrosis, but PPARγ signaling induces the opposite conversion, which mediates the resolution of fibrosis [239]. Zabihi et al. also noted, on the basis of an aged mouse model of delayed resolution, that the transformation of lipofibroblast into myofibroblast can be reversed at the stage of fibrosis resolution. Moreover, ADAMTS4^+^ alveolar fibroblasts were maintained throughout the repair phase, indicating that the ADAMTS4/VCAN axis is a major mechanism behind the delayed resolution of fibrosis [240]. Liu et al. showed with integrated single-cell sequencing and primary cell functional experiments that both lipofibroblasts and Ebf1^+^ fibroblasts directly contribute to matrix deposition. Both subpopulations displayed increased invasive characteristics under profibrotic stimulation, and Ebf1^+^ fibroblasts also exhibited considerable contractile ability [241]. Collectively, these results indicate that stromal fibroblast subpopulations, which are traditionally considered to be quiescent, may develop pathological effector functions upon exposure to profibrotic microenvironments.

#### 4.4.2. Fibroblast Activation Regulatory Network

The TGF-β/Smad axis has long been considered the master switch of fibroblast activation: after TGF-β1 binds to its receptor, Smad2/3 is phosphorylated and forms a complex with Smad4 that translocates into the nucleus to initiate transcription of target genes, including COL1A1 and α-SMA [19,145]. Nevertheless, this axis does not act alone, and recent research has shown that there are several parallel or intersecting tracks of signaling.

In Smad-independent bypass pathways, there are several signaling tracks that ultimately converge onto shared downstream nodes. P61-Sema3E is a profibrotic cleavage product of Semaphorin 3E that is enriched in the plasma and lung tissues of IPF patients. It stimulates Plexin D1-ErbB2 receptor complex activation and AKT/ERK phosphorylation without causing Smad2/3 phosphorylation [242]. In the same manner, CCL1 induces AMFR-mediated K27-linked ubiquitination of Spry1 and its translocation to the membrane, thus alleviating Spry1-RasGAP inhibition of the Ras-ERK-p70S6K axis and promoting fibroblast-to-myofibroblast transition [243]. Although these two pathways have different receptors of origin, they both lead to ERK, indicating that this kinase may be a typical mechanism that drives fibroblast activation. In the meantime, PAK2 serves as a non-canonical downstream kinase of TGF-β, selectively activated in pathological fibroblasts in fibrotic foci and dense fibrotic areas, with expression levels significantly higher than PAK1/PAK3 [89], suggesting that fibroblasts in various microenvironments have different kinase preferences.

Some signals augment the classical axis by cooperative processes. WNT5A fails to directly activate TGF-β receptors or phosphorylate Smad, but rather, rapidly rearranges the cytoskeleton through JNK/ROCK signaling to cluster integrin αV and produce mechanical tension to release latent TGF-β and indirectly stimulate Smad3 activation [244]. Moreover, post-transcriptional regulation enhances the TGF-β/Smad pathway: the RNA-binding protein Rbms1 stabilizes Sumo2 mRNA, which promotes SUMOylation of Smad4 at K158 and thus boosts its profibrotic transcriptional program [245]. Conversely, the PARP1-FOXN3 axis has been implicated as an endogenous brake on Smad transcriptional activity. PARP1 appears to sustain FOXN3-mediated repression of Smad signaling by stabilizing FOXN3 and limiting its p38-mediated degradation, whereas loss of either protein enhances Smad binding to profibrotic target genes and aggravates fibrosis in mice; both proteins are markedly reduced in fibrotic lesions from patients with pulmonary fibrosis [246].

Epigenetic changes stably commit the cell to an activated state after signal activation. MBD2 binds to methylated sites induced by TGF-β1 in the Erdr1 promoter and represses this negative regulator, thus removing inhibition of the TGF-β/Smad pathway and constantly driving profibrotic signals [247]. UHRF1, by methylating the Beclin1 promoter via Dnmt1, inhibits autophagy and promotes fibroblast activation [248]. These factors synergistically lock the activated phenotype at different genetic loci. Enzymes that modify histones and their cofactors are highly specialized. DOT1L mediates H3K79 trimethylation at the Jag1 promoter to activate Notch signaling and induce ECM production [249]. Meanwhile, CBX5/G9a-mediated H3K9 methylation represses PGC1α, leading to mitochondrial dysfunction and persistent activation of fibroblasts [250]. KMT2A catalyzes H3K4 trimethylation at the PU.1 promoter to promote profibrotic gene expression programs and fibroblast activation [251]. These three complexes coordinate distinct sets of profibrotic gene expression programs to set up the epigenetic code for fibroblast activation.

HDACs have cell-type-specific regulatory roles. HDAC3 deacetylates NICD1 and STAT1 in epithelial cells and fibroblasts, leading to activation of Notch1 and STAT1 [252]. In hypoxia, HDAC3 facilitates alveolar epithelial-to-mesenchymal transition and migration of fibroblasts via the AKT-HIF-1α axis [253]. In contrast, PHLPP1-mediated HDAC8 activation in alveolar macrophages decreases KLF4 promoter acetylation, thereby suppressing fibrosis [254]. Conversely, decreased histone acetylation at the Fas promoter in lung fibroblasts from fibrotic tissue renders them resistant to apoptosis, which represents another profibrotic consequence of altered histone acetylation [255].

A more important problem than the signal maintenance is the self-reinforcing loop between metabolism and signaling. TGF-β1 causes PFKFB3-mediated glycolytic reprogramming in fibroblasts [256], and Akt2-PDK1 axis further stimulates lactate production [257]. Recent research has shown that lactate not only acts as a metabolic substrate but also directly stabilizes TGF-β1, as lactate-induced H3K18 lactylation suppresses CHIP expression in macrophages, thereby impeding TGF-β1 degradation and enhancing its secretion [258]. The metabolism of glutamine is involved in this self-amplifying loop: TGF-β1 induces the expression of GLS1 [259], and the product of the reaction (α-ketoglutarate) facilitates H3K27 trimethylation demethylation to regulate the transcription of profibrotic genes [260]. These mechanisms are further interrelated with histone lactylation to form an entire closed loop: H3K18 lactylation upregulates YTHDF1, which promotes m6A-dependent NREP mRNA degradation [227], and at the same time, causes METTL3-mediated PGC-1α mRNA degradation and mitochondrial dysfunction [261]. Activated myofibroblasts exhibit mainly glycolytic phenotypes and produce lactate continuously [256,262], thus sustaining the above profibrotic program.

### 4.5. ECM Remodeling and Mechanical Microenvironment

Collagen, elastin, fibronectin, proteoglycans and laminin are the components of normal lung ECM that give mechanical support and elastic recoil to alveolar structures and control cell adhesion, migration, proliferation and differentiation via cell–matrix interactions. In IPF, both the composition and structure of the ECM undergo dual remodeling: matrix proteins (collagens, elastin, fibronectin, and proteoglycans) are significantly deposited, whereas collagen cross-linking by the lysyl oxidase (LOX) family is increased, progressively increasing tissue stiffness. Moreover, imbalance in matrix metalloproteinases (MMPs) and their tissue inhibitors of metalloproteinases (TIMPs) leads to impaired degradation of ECM which further aggravates deposition of matrices [263,264,265]. The conventional opinion is that changes in ECM are secondary effects of the activation of fibroblasts. Nevertheless, pathological ECM is not a passive structural deposit but an active signaling platform that controls cellular behavior—Parker et al. showed that pathological ECM itself can program normal fibroblasts to adopt a profibrotic phenotype by translational reprogramming and create a positive feedback loop by suppressing miR-29 [266]. Moreover, LOXL2-mediated stimulation of collagen cross-linking enhances mechanical tension in the matrix, which subsequently activates latent TGF-β1 and constantly stimulates the activation of fibroblasts [264]. This dynamic remodeling can be tracked in real-time using serum neoepitope markers, with baseline values of collagen turnover fragments like PRO-C3 and C1M being strongly correlated with disease progression and risk of mortality in IPF [265,267]. This implies that the evaluation of fibrosis is shifting towards biological monitoring as opposed to static imaging.

Mechanosensing, through integrin-based focal adhesions, translates matrix stiffening into intracellular profibrotic signals. Integrin engagement recruits and activates FAK, whose autophosphorylation at Tyr397 creates a docking site for Src family kinases; the FAK–Src complex subsequently activates PI3K–AKT and Ras–MAPK/ERK signaling, regulating cytoskeletal organization, proliferation and fibroblast survival [268]. Accordingly, FAK inhibition suppresses fibroblast activation and collagen deposition in experimental lung fibrosis, identifying focal adhesion signaling as a central mechanosensing hub. Beyond sensing stiffness per se, integrins also mediate the mechanical release of latent mediators: integrin αvβ6 on epithelial cells binds to the latency-associated peptide (LAP) of TGF-β1 and, through cytoskeletal force generation, liberates active TGF-β1 [269].

The increased stiffness of the ECM not only activates fibroblasts but may also alter epithelial fate and ECM architecture via mechanosignals. Single-cell sequencing experiments have revealed that pathological epithelial cells with a KRT5^−^/KRT17^+^ phenotype are mainly found in fibrotic niches in lung tissue from IPF patients, and express ECM-related genes including COL1A1, FN1 and ITGB6 [20]. At the same time, airway basal cells from IPF patients are reprogrammed to a dedifferentiated state marked by high levels of KRT17 and low levels of PTEN, and are enriched in pathways associated with ECM sensing, focal adhesion and actin cytoskeleton regulation [270]. Taken together, these studies suggest that an aberrant mechanical microenvironment may activate basal cell lineages and their differentiated populations toward a profibrotic phenotype, thus representing a potential key step in the link between mechanical signals and epithelial disease.

The yes-associated protein (YAP) and transcriptional coactivator with PDZ-binding motif (TAZ), as mechanosensitive coactivators, translate matrix stiffening signals into profibrotic transcriptional programs to activate, proliferate and secrete excessive matrices in fibroblasts [271,272]. In the nucleus, stiffness-activated YAP/TAZ is associated with TEAD transcription factors to induce a profibrotic transcriptional program, including CTGF, CYR61 and ECM genes, thereby activating fibroblasts to proliferate and secrete the excessive matrix [271,272,273]. Mechanosensing upstream of YAP/TAZ is not restricted to integrin-focal adhesion signaling. The mechanosensitive ion channel PIEZO1 transduces mechanical stretch into YAP/TAZ activation in periostin-positive myofibroblasts, and myofibroblast-specific deletion of Yap/Taz attenuates myofibroblast accumulation and lung fibrosis in mice [274]. Together, these processes create a vicious cycle: extracellular matrix deposition–tissue stiffening–YAP/TAZ nuclear translocation and activation of transcription–more matrix deposition. Thus this cycle perpetuates the fibrotic response.

These ECM remodeling and cell-type changes are not randomly distributed, but are spatially grouped into functional niches with different cellular compositions and programs. By applying Visium and Xenium spatial transcriptomics, researchers found three disease-specific niches in IPF lung tissue: a fibrotic niche, consisting of CTHRC1^+^ myofibroblasts and KRT5^−^/KRT17^+^ aberrant basaloid cells, which was significantly enriched in ECM-associated pathways; an airway macrophage niche (SPP1^+^ macrophages); and an immune niche (B/T cells and PLVAP^+^ ectopic endothelial cells) [131]. Another study independently confirmed the spatial organization of these pathologic cellular communities using subcellular-resolution Xenium data on a larger cohort [132]. Lingampally et al. also demonstrated that mesenchymal cells that make up these niches have a high level of phenotypic plasticity: lipofibroblasts and Cthrc1^+^ myofibroblasts can be converted into each other during murine lung fibrosis, but this reverse conversion is limited in human IPF, and the activity of alveolar mesenchymal niches cannot be restored during resolution of fibrosis [275]. All these observations indicate that the ECM features of fibrotic foci are not the result of one niche acting alone, but are instead an emergent phenomenon due to the coordinated interplay of many niches and their respective internal cellular programs.

### 4.6. Critical Appraisal of scRNA-Seq and Spatial Multi-Omics Evidence

The advent of scRNA-seq and spatial multi-omics technologies has provided a strong basis for the detailed analysis of pathological cell populations in pulmonary fibrosis. However, the information derived from single-cell and other omics data needs to be interpreted with discrimination. Tissue dissociation induces stress-response genes and preferentially damages fragile epithelial and endothelial cells, so the abundance of rare populations such as aberrant basaloid cells and CTHRC1^+^ fibroblasts reported by scRNA-seq may not accurately reflect their true proportions in vivo [276,277]. Similar caveats apply to the spatial claims in the literature, as scRNA-seq destroys spatial information by design, and although the enrichment of these populations in fibroblastic foci or perilesional regions has been confirmed by in situ hybridization and immunostaining, transcriptome-wide spatial mapping at fine resolution still depends on platforms that either average transcripts across multiple cells or cover only a limited set of genes, with cell-type assignment in part computationally inferred rather than directly observed [36,39,131,132,278]. Interpretation is further complicated by the nature of the available samples and data. Most human fibrotic lung tissues analyzed to date come from end-stage explanted lungs or late-stage biopsies and therefore poorly represent the early events of fibrosis initiation [20,36], and batch differences in tissue handling and sequencing together with inconsistent naming of transitional cell states complicate comparison across studies [102,279,280]. The dynamic processes reconstructed from these data should also be read as models rather than observations, because transitional states and differentiation trajectories are inferred from static snapshots using pseudotime or RNA-velocity analysis whose kinetic assumptions are difficult to verify in remodeling tissue [281,282]. Most importantly, the ligand–receptor interaction networks and the profibrotic roles attributed to specific cell populations remain correlational in human tissues, resting on expression patterns and spatial proximity. Although several of these pathways have been validated by perturbation experiments such as lineage tracing and conditional knockout, most of this validation has been performed in mouse models, particularly bleomycin-induced fibrosis, whose reversibility and incomplete fidelity to human disease limit the causal conclusions that can be drawn [53,283]. These results therefore serve as a valuable guide to understanding the cellular processes underlying pulmonary fibrosis, but their causal role in human disease needs to be validated by functional and clinical studies before they can be used to support mechanistic inferences and therapeutic strategies.

## 5. Advances in Pulmonary Fibrosis Therapy

### 5.1. Current Therapeutic Landscape and Unmet Needs

Pirfenidone and nintedanib are the standard antifibrotic therapies for IPF. The ASCEND and INPULSIS trials demonstrated that both agents significantly slow the decline in FVC and delay disease progression. However, neither can reverse established pulmonary fibrosis or restore lung architecture [284,285]. Nerandomilast (BI 1015550), the PDE4B inhibitor, exerts anti-inflammatory and antifibrotic effects through selective inhibition of the PDE4B. A phase 2 study showed that 18 mg twice daily stabilized lung function over 12 weeks in patients with IPF [286]; the phase 3 FIBRONEER-IPF trial further demonstrated that at week 52, the adjusted mean change in FVC was −114.7 mL in the 18 mg group, representing a significant reduction of 68.8 mL compared with the placebo (*p* < 0.001), with robust efficacy maintained when used in combination with background antifibrotic therapy [287]. This agent has received FDA approval for progressive pulmonary fibrosis (PPF). Inhaled treprostinil modulates profibrotic signaling through activation of prostaglandin receptors (EP2, DP1, and PPAR), and inhaled delivery enables high drug deposition at the alveolar interface. The TETON-2 phase 3 trial showed that after 52 weeks of inhaled treprostinil, patients with IPF had a median FVC decline of 49.9 mL, which is significantly less than the 136.4 mL observed with the placebo (between-group difference of 95.6 mL, *p* < 0.001), and a 29% lower risk of clinical worsening (HR 0.71, 95% CI 0.53–0.95; *p* = 0.02) [288]. Rentosertib, the TNIK inhibitor, inhibits fibroblast activation by blocking Wnt/β-catenin and TGF-β signaling. In a phase 2a trial, the mean increase in FVC from baseline after 12 weeks of treatment with 60 mg rentosertib was 98.4 mL in patients with IPF, compared to a decrease of 20.3 mL in those treated with placebo [289]. Deupirfenidone, the deuterated form of pirfenidone, has an improved pharmacokinetic profile due to selective deuteration while maintaining inhibition of TGF-β and TNF-α pathways. In the ELEVATE-IPF phase 2b trial, the 825 mg three-times-daily (TID) dose showed a mean change in FVC of −21.5 mL at week 26, compared to −112.5 mL with placebo (*p* = 0.02), thus achieving the primary endpoint [290]. These promising findings have led to a global phase 3 trial (SURPASS-IPF, NCT07284602) to further assess deupirfenidone. Current treatments primarily target downstream fibrotic processes, with little targeting of upstream factors such as epithelial damage and immune microenvironment abnormalities. New approaches targeting these factors are becoming increasingly prominent.

### 5.2. Targeting Alveolar Epithelial Injury and Aberrant Repair

As discussed above, aberrant repair and AT2 cell senescence are key processes in the initiation of pulmonary fibrosis. Yet clinical data suggest that clearance of senescent cells is not sufficient to rapidly reverse fibrotic lung architecture. Although the senolytic combination of dasatinib and quercetin (D + Q) was well-tolerated in an open-label pilot study [291] and a subsequent randomized, placebo-controlled pilot trial [292] in IPF patients, there was no significant improvement in pulmonary function (FVC and FEV1) after 3 weeks of treatment, indicating that the focus should be shifted from “senescent cell clearance” to repair strategies. Senomorphics represents an alternative anti-aging strategy alongside senolytics. Unlike senolytics, which kill senescent cells, senomorphics reduce SASP by targeting pathways like NF-κB, thus changing the “behavioral pattern” of senescent cells [293]. For instance, resveratrol reduces bleomycin-induced pulmonary fibrosis in mice by repressing SASP factors such as SERPINE1, MMP2 and IL-6, thereby inhibiting AT2 cell senescence and extracellular matrix deposition [294].

Approaches to AT2 regeneration and repair are mostly in the early investigation stage. The modified DPP4 inhibitor, CMR316, stimulates healing by stimulating alveolar epithelial stem cells, and its phase I trial began in 2024 (NCT06589219). In the nucleic acid delivery field, inhalable mucus-penetrating lipid nanoparticles (LNPs) for codelivering CYB5R3 and BMP4 mRNAs, which in a bleomycin-induced mouse model reverse AT2 mitochondrial dysfunction by ameliorating NAD^+^ biosynthesis, inhibit accelerated senescence of AT2 cells and ultimately prompt alveolar regeneration [295]. In terms of cell therapy, Alvarez-Palomo et al. grafted human iPSC-derived alveolar type II-like cells into a rat fibrosis model, which showed much less collagen deposition and better lung alveolar structure. It is noteworthy that the differentiated cells expressed surfactant protein C and other markers characteristic of AT2 in vitro [296]. Although these preclinical developments are promising, there are still several challenges to clinical translation of AT2 regenerative strategies in terms of long-term efficacy, safety, and large-scale production.

Beyond senescence-directed strategies, epithelial death pathways have also entered clinical development. RIPK3-mediated necroptosis has been directly documented in alveolar epithelial cells, in which RIPK3 is upregulated in IPF lungs and necroptotic death occurs mainly in AECs, releasing DAMPs such as HMGB1 and IL-1β that amplify inflammation and fibrosis, whereas necrostatin-1 and RIPK3 knockout attenuate bleomycin-induced fibrosis in mice [297]. SFTPA1 mutation promotes JNK-dependent RIPK3 activation and necroptosis in AT2 cells, and JNK inhibition ameliorates fibrosis in surfactant-mutation knock-in mice [298]. The c-Jun N-terminal kinase inhibitor CC-90001 reduced the 24-week decline in percent predicted by FVC in a dose-dependent manner in a phase 2 trial in IPF patients, accompanied by reductions in circulating biomarkers of collagen synthesis and extracellular matrix remodeling, although the between-group differences did not reach statistical significance [299].

### 5.3. Targeting Pulmonary Endothelial Injury and the Vascular Microenvironment

EndMT is an important step between the disruption of pulmonary vascular microenvironment and fibrosis amplification. Mangiferin has been shown to potently suppress EndMT and perivascular fibrosis in diabetic models through activation of the AMPK/FoxO3/SIRT3 pathway, but there are no clinical data on this approach yet [300]. On the other hand, the phase 2 trial of the second-generation LPA1 antagonist, BMS-986278 (admilparant), enrolled parallel cohorts of patients with IPF and PPF, aiming to intervene in fibrotic progression at the interstitial–vascular interface by blocking LPA1-mediated vascular leakage and fibroblast recruitment [301]. Results showed that the 60 mg dose slowed lung function decline over 26 weeks (treatment difference in ppFVC: 3.2% in the PPF cohort and 1.4% in the IPF cohort) and was well-tolerated [302]. In summary, therapeutic strategies targeting the pulmonary vascular microenvironment have demonstrated clear antifibrotic potential, and future studies are needed to further validate their long-term efficacy.

### 5.4. Targeting Macrophage Remodeling and Reprogramming

Macrophage targeting is shifting from non-selective depletion toward precise state modulation. CSF1R inhibitors attenuate myofibroblast-driven processes by blocking profibrotic macrophage activation and the release of mediators such as TGF-β. In the AGAVE-201 trial, axatilimab (0.3 mg/kg every two weeks) achieved a 74% response rate in refractory chronic graft-versus-host disease (GVHD), with responses observed in fibrotic organs including the lungs and skin; this low dose induced only transient reductions in non-classical monocytes without elevating CSF1, better preserving immune homeostasis than sustained depletion [303]. The FDA has approved it for refractory GVHD, and a phase II MAXPIRe trial (NCT06132256) is currently evaluating its efficacy in idiopathic pulmonary fibrosis (IPF). In animal models of pulmonary fibrosis, pexidartinib combined with nintedanib significantly reduced M2 macrophages and promoted M1 phenotypic transition by modulating macrophage polarization, synergistically reversing fibrosis [304]. In recent years, researchers have developed various nanomedicine strategies that target or harness macrophages. For example, the nanocarrier siOPN@LuZ-M2, constructed from a luteolin–Zr metal–polyphenol network conjugated with an M2-targeting peptide, enables targeted delivery of OPN siRNA to M2 macrophages, thereby suppressing their profibrotic polarization via OPN silencing [305]. The M/RH-L system, which employs RAW264.7 macrophages as carriers, can chemotax to fibrotic regions and release its payload via extracellular traps, synergistically upregulating autophagy and inhibiting epithelial-to-mesenchymal transition (EMT) to restore alveolar structure [306]. At the functional reprogramming level, nobiletin enhances macrophage autophagy via activation of the AMPK-mTOR axis, thereby suppressing M2 polarization and the release of profibrotic mediators (e.g., TGF-β and CTGF) [307]. From a metabolic reprogramming perspective, the endogenous metabolite itaconate and its derivatives exert anti-inflammatory and antifibrotic effects in bleomycin-induced pulmonary fibrosis models by inhibiting succinate dehydrogenase and the NLRP3 inflammasome, while concurrently activating the Nrf2 and ATF3 anti-inflammatory transcriptional axes [308]. In the context of nucleic acid therapeutics, intratracheally administered Mbd2 or Sart1 siRNA-loaded liposomes efficiently accumulate in pulmonary fibrotic lesions and are taken up by macrophages, thereby suppressing the M2 program by blocking the Ship/PI3K/Akt and STAT6/PPAR-γ signaling axes, respectively, and significantly alleviating pulmonary fibrosis [309,310].

### 5.5. Targeting Profibrotic Signaling, Fibroblast Activation, and ECM Remodeling

Fibroblast activation is a critical executive step in the fibrotic cascade. TNIK (TRAF2- and NCK-interacting kinase) integrates multiple profibrotic signals to regulate fibroblast activation and ECM production. Rentosertib is the world’s first AI-designed TNIK inhibitor, which significantly suppresses fibroblast activation in preclinical models [311]. In a phase 2a trial, the 60 mg group exhibited an FVC increase of 98.4 mL at 12 weeks (versus a 20.3 mL decline in the placebo group), with concurrent reductions in serum COL1A1, FAP and other biomarkers [289]. If its efficacy is confirmed, this drug could mark a milestone in AI-empowered antifibrotic therapy. The dual αvβ6/αvβ1 integrin inhibitor Bexotegrast (PLN-74809) reduces pulmonary type I collagen deposition by blocking localized TGF-β activation mediated by epithelial cells and fibroblasts. In the phase 2 trial, 12 weeks of treatment decreased the lung collagen PET signal by 20%, and dose-dependently reduced FVC decline with improved cough severity in INTEGRIS-IPF [312,313]. IL-11 is a crucial downstream executor of TGF-β signaling. Schafer et al. found that IL-11 is markedly upregulated in fibroblasts in response to TGF-β1 stimulation, where it drives fibroblast activation and fibrogenic protein synthesis via non-canonical ERK (extracellular signal-regulated kinase)-dependent autocrine signaling. Based on this, genetic deletion of Il11ra1 attenuates fibrosis in cardiac and renal fibrosis models [314]. Inhaled siRNA nanoparticles targeting IL-11 have significantly attenuated fibrosis and improved pulmonary function in a bleomycin-induced pulmonary fibrosis animal model [315]. Hedgehog signaling drives α-SMA transcription via the SHH-Sm-Gli axis and establishes a TGF-β1 positive-feedback loop. In a phase 2a trial of IPF patients, the Smo inhibitor taladegib increased FVC by 3.95% and TLC by 257 mL compared to the placebo [316]. WISP1 (WNT1-inducible signaling pathway protein 1), a downstream effector of TGF-β1, stimulates fibroblast activation and ECM deposition through integrin α5β1; its neutralizing antibody MTX- 463 has already entered a phase 2a trial (NCT06967805). PROTAC-mediated targeted degradation of BRD4 inhibits fibroblast activation and ECM remodeling in preclinical models [317]. In an open-label phase 2 trial in IPF patients, patients treated with the selective ROCK2 inhibitor belumosudil had a median FVC increase of 50 mL at week 24, whereas those receiving the best supportive care had a median decline of 175 mL (NCT02688647), and the next-generation ROCK2 inhibitor zelasudil is undergoing a phase 2 evaluation (NCT05570058) (Table 1).

These multidimensional therapeutic approaches across signaling nodes, integrin blockade, cytokine intervention, and protein degradation are moving beyond mechanistic studies to early clinical validation and are providing an ever more obvious translational roadmap to precisely target fibroblast activation and ECM remodeling.

### 5.6. Emerging Strategies and Translational Challenges

Alongside the above mechanism-focused approaches, several avenues of research are moving from bench to bedside.

#### 5.6.1. Epithelial Injury and Repair

New cell death mechanisms provide a wider target. Ferroptosis is iron-dependent lipid peroxidation. Elevated iron and reduced glutathione peroxidase 4 (GPX4) antioxidant activity are found in the lungs of IPF patients. In bleomycin models, the ferroptosis blocker Liproxstatin-1 and iron chelator deferoxamine (DFO) ameliorate epithelial cell damage and collagen deposition, offering novel targets for antioxidant therapy [318,319]. Copper homeostasis has recently emerged as an additional epithelial target. Bleomycin injury elevates lung copper and oligomerized DLAT in vivo, and copper ionophores trigger cuproptosis in lung epithelial cells, whereas the copper chelator tetrathiomolybdate normalizes copper levels, attenuates oxidative stress and cuproptosis, and mitigates TGF-β-driven EMT and fibroblast-to-myofibroblast transition in bleomycin models [320]. Given that tetrathiomolybdate is already used clinically for copper metabolism disorders, copper chelation represents a repurposing-ready strategy in this compartment. Senescence clearance is also being refined at the preclinical level. Fisetin, a natural flavonoid with senolytic activity, alleviates bleomycin-induced pulmonary fibrosis partly by rescuing alveolar epithelial cells from senescence through AMPK activation and suppression of TGF-β/Smad3 signaling [321].

#### 5.6.2. Immune Modulation

Gut microbiota composition critically affects pulmonary fibrosis. Variations in gut microbiota between mice from different vendors directly dictate survival and fibrosis in bleomycin-treated models, while horizontal transfer of microbiota by fecal microbiota transplantation (FMT) or cohousing alters intestinal microecology, induces pulmonary regulatory immunity and reduces lung injury [322], suggesting the adjunctive potential of probiotics and nutrition. However, there are currently no clinical trials in IPF patients. Many natural product monomers also show antifibrotic activity by directly targeting critical signaling pathways. Berberine improves the pulmonary inflammatory microenvironment and decreases collagen deposition in bleomycin-induced models by suppressing MAPK/p38 signaling, thereby increasing model survival [323]. Lycorine ameliorates bleomycin-induced pulmonary fibrosis by blocking NLRP3 inflammasome activation and downstream pyroptosis [324]. Likewise, network pharmacology and docking analyses have suggested that Astragalus polysaccharides may target multiple nodes of TNF, NF-κB and MAPK signaling implicated in pulmonary fibrosis, but such in silico predictions await experimental and clinical validation [325]. Thus, most of these natural product studies still remain at the preclinical stage, with complex formulations and unknown pharmacokinetics, requiring further clinical validation. Drug repurposing and innate immune targets further expand this compartment. The FDA-approved antiparasitic agent ivermectin suppresses NLRP3 inflammasome activation and ameliorates bleomycin-induced pulmonary fibrosis in rats [326], while the mitochondria-targeted agent NecroX-5 inhibits NLRP3-mediated epithelial–mesenchymal transition and fibrosis [327].

#### 5.6.3. Fibroblast Activation, ECM Remodeling and Mechanotransduction

Triptolide reduces ECM remodeling by blocking integrin-β1–focal adhesion kinase–YAP mechanotransduction signaling [328]. Pharmacological inhibition of the same machinery is being actively explored, as the FAK inhibitors defactinib and PF-562271 suppress TGF-β-driven fibroblast activation in IPF-derived cells in vitro and attenuate bleomycin-induced lung fibrosis in vivo [329,330]. Notably, defactinib has accumulated an acceptable safety profile across phase 1 to 3 oncology trials, raising the possibility that FAK inhibition could be repurposed for pulmonary fibrosis once antifibrotic efficacy is confirmed in patient-relevant models. Mechanosensitive ion channels have recently entered this landscape, as myofibroblast-specific PIEZO1 deletion and its pharmacological blockade by GsMTx4 both attenuate bleomycin-induced fibrosis and improve survival through suppression of YAP/TAZ nuclear translocation [274]. However, all current evidence remains preclinical.

#### 5.6.4. Innovation in Drug Carriers

**Figure 4:**
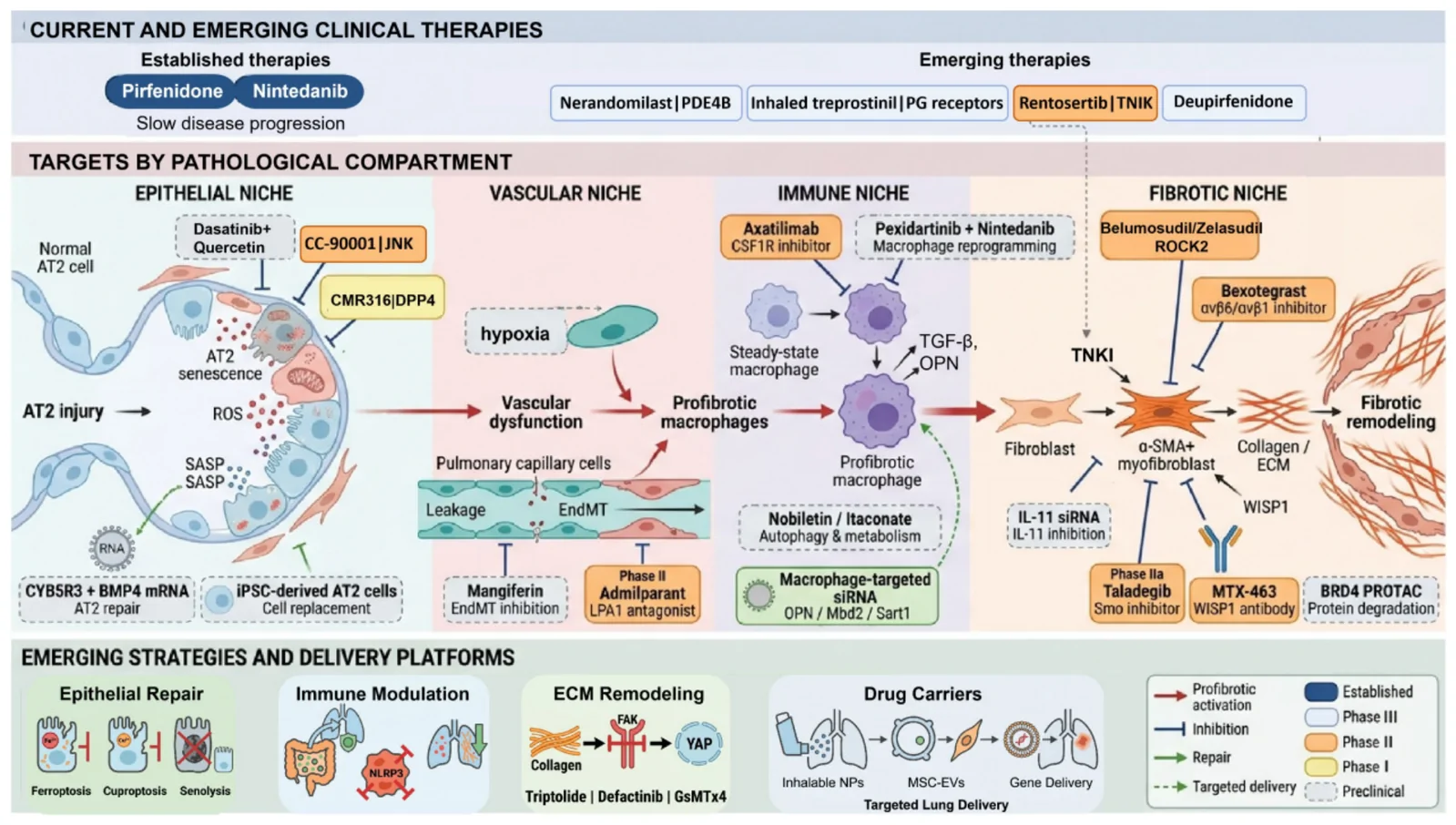
Mechanism-targeted therapies and their clinical translation in pulmonary fibrosis. This figure encapsulates existing antifibrotic drugs, such as pirfenidone and nintedanib, and new approaches targeting epithelial repair, vascular and immune microenvironments, profibrotic signaling, fibroblast activation and ECM remodeling. Gut–lung axis modulation and ferroptosis inhibition offer additional avenues. Natural compounds show promise. The use of extracellular vesicles and inhaled nanoparticles as delivery vehicles provides novel approaches for targeted intervention and even reversal of pulmonary fibrosis.

The combination of natural products and nano-delivery systems has greatly improved the local targeting and delivery efficiency of conventional active ingredients to fibrotic lesions. Inhalable luteolin, hyaluronidase nanoparticles that can effectively penetrate deep into tissues by degrading interstitial hyaluronic acid, are preferentially taken up at IPF lesions; they scavenge ROS and suppress α-SMA expression more efficiently than free drugs [331]. An inhalable nano-platform loaded with astragaloside IV and tetramethylpyrazine inhibits NOX4/NLRP3 signaling in a dual manner, simultaneously targeting early inflammatory and late fibrotic stages [332]. EVs are a new innovation in drug carriers. Exosomes derived from mesenchymal stem cells (MSCs) are taken up by Thy-1-mediated endocytosis through binding to β-integrins, which suppresses TGF-β1-induced myofibroblast differentiation. They are non-immunogenic and can be genetically modified, but long-term safety and scalable manufacturing are challenges for their clinical use [333,334]. Non-viral nano-delivery systems (liposomes and polymeric nanoparticles) are enriched locally in the lungs via inhalation, enhancing the concentration of drugs at the lesion site and minimizing systemic exposure, thus offering an effective and safe delivery system for IPF gene therapy [335] (Figure 4).

These frontier approaches (intestinal microecological regulation, novel cell death intervention and advanced drug delivery system) have significantly broadened the therapeutic horizon of pulmonary fibrosis at a mechanistic level. But their development from bench to bedside is limited by systematic challenges such as extrapolation of animal studies, standardization of formulations, and long-term safety, which calls for patient-focused rigorous clinical trials to connect basic science with clinical practice.

## 6. Conclusions and Future Perspectives

Pulmonary fibrosis is caused by abnormal repair. It should no longer be viewed as a disorder driven by the aberrant activation of individual signaling pathways, but rather as a dynamic, multicellular process in which multiple cell types continuously interact with and reinforce one another in a spatially and temporally evolving microenvironment. This paradigm shift calls for a new approach to disease management. The common pathological microenvironment underlying idiopathic pulmonary fibrosis, progressive pulmonary fibrosis and cancer therapy-related lung injury demonstrates that although these diseases are initiated by different factors, they converge on a common pathway: aberrant epithelial regeneration, endothelial barrier dysfunction, which leads to local hypoxia, fibroblast activation, macrophage reprogramming, which drives profibrotic immune responses, and progressive ECM stiffening, which establishes a vicious cycle of mechanical feedback. Single-cell and spatial transcriptomics have uncovered key cell types in this milieu: abnormal KRT5^−^/KRT17^+^ epithelial cells, SPP1^+^/TREM2^+^ macrophages, and CTHRC1^+^ fibroblasts. Because these populations and pathways exist within this spatially and temporally evolving cellular ecosystem, their causal roles cannot be inferred from static snapshots alone. But the discovery of these pathological cell types is just the beginning. The big question is why normal healing processes go wrong and how aberrant signals become trapped in vicious cycles.

Current antifibrotic therapies have achieved moderate success, but their inability to reverse fibrosis suggests a major gap: current treatments target the symptoms of failed repair rather than its causes. In the future, combining multi-omics with mechanobiology to fully define the pulmonary fibrosis microenvironment will help determine how epithelial barrier dysfunction, immune dysregulation and mechanical stiffening are integrated, allowing the identification of specific nodes where repair has failed. This involves changing the therapeutic strategy from “inhibiting profibrotic signals” to “restoring tissue repair”. However, our current knowledge of these processes remains largely inferential, derived from static snapshots and animal models. Before it can guide the development of corrective therapies, candidate cells and pathways must be tracked longitudinally and mapped at spatial resolution across disease stages, verified in human lung tissue from well-characterized patient cohorts, and tested through targeted perturbation experiments that establish causality rather than mere association. Because fibrotic signaling is redundant and highly interconnected, clinically relevant combination therapies that simultaneously target multiple nodes of this microenvironment will likely be required to disrupt the vicious cycle of pathology. Ultimately, the key to defeating pulmonary fibrosis is not the fine-tuning of existing drugs, but a paradigm shift in our approach to this disease—recognizing it as an aberrant repair process that can be halted by targeting the common pathological microenvironment.

## Figures and Tables

**Table 1:** Representative clinical trials of pathogenesis-targeted agents in PF.

Pathological Pattern	Targets	Agents	Diseases	Phase	NCT
Epithelial injury	Senescent cells	Dasatinib + Quercetin	IPF	phase I trial	NCT02874989
	DPP4	CMR316	IPF	phase I trial	NCT06589219
	JNK	CC-90001	IPF	phase II trial	NCT03142191
Endothelial dysfunction	LPA1	Admilparant	IPF; PPF	phase II trial	NCT04308681
Macrophages	CSF-1R	Axatilimab	IPF	phase II trial	NCT06132256
Fibroblast, ECM and mechanotransduction	TNIK	Rentosertib	IPF	phase II trial	NCT05938920
	αvβ6/αvβ1	Bexotegrast	IPF	phase II trial	NCT04396756
	Smo	Taladegib	IPF	phase II trial	NCT04968574
	WISP1	MTX-463	IPF	phase II trial	NCT06967805
	ROCK2	Belumosudil	IPF	phase II trial	NCT02688647
	ROCK2	Zelasudil	IPF	phase II trial	NCT05570058
Others	PDE4B	Nerandomilast	IPF	phase III trial	NCT05321069
	PG receptor	Inhaled treprostinil	IPF	phase III trial	NCT05255991
	Nonspecific	Deupirfenidone	IPF	phase II trial	NCT05321420

## Data Availability

No new data were created or analyzed in this study. Data sharing is not applicable to this article.

## Supplementary Materials

The following supporting information can be downloaded at https://www.mdpi.com/article/10.3390/biomedicines14102265/s1, Table S1: Search strings used in PubMed.
