# Supplementary material for: The Shared Pathological Niche in Pulmonary Fibrosis: From Aberrant Epithelial Repair to ECM Remodeling
Source: Biomedicines. 2026 Oct 7;14(10):2265. doi: 10.3390/biomedicines14102265 (PMC13701973; doi:10.3390/biomedicines14102265)
Supplement: Supplementary file 1 [file biomedicines-14-02265-s001.zip › biomedicines-4566811-supplementary.pdf]

Supplementary Table S1. Search strings used in PubMed

Search string	("Idiopathic Pulmonary Fibrosis"[Mesh] OR "idiopathic pulmonary fibrosis"[Title/Abstract] OR "pulmonary fibrosis"[Title/Abstract] OR "lung fibrosis"[Title/Abstract] OR "interstitial lung disease"[Title/Abstract] OR "usual interstitial pneumonia"[Title/Abstract] OR "idiopathic interstitial pneumonia"[Title/Abstract] OR "radiation-induced lung injury"[Title/Abstract] OR "radiation pneumonitis"[Title/Abstract] OR "radiation-induced pulmonary fibrosis"[Title/Abstract] OR "drug-induced pneumonitis"[Title/Abstract] OR "drug-induced interstitial lung disease"[Title/Abstract] OR "hypersensitivity pneumonitis"[Title/Abstract] OR "silicosis"[Title/Abstract] OR "asbestosis"[Title/Abstract] OR "pneumoconiosis"[Title/Abstract] OR ("systemic sclerosis"[Title/Abstract] AND "interstitial lung disease"[Title/Abstract]) OR ("rheumatoid arthritis"[Title/Abstract] AND "interstitial lung disease"[Title/Abstract]) OR (("COVID-19"[Title/Abstract] OR "post-COVID-19"[Title/Abstract] OR "post-COVID"[Title/Abstract]) AND ("pulmonary fibrosis"[Title/Abstract] OR "lung fibrosis"[Title/Abstract]))) AND ("single-cell"[Title/Abstract] OR "scRNA-seq"[Title/Abstract] OR "spatial transcriptomics"[Title/Abstract] OR "macrophage"[Title/Abstract] OR "neutrophil"[Title/Abstract] OR "fibroblast"[Title/Abstract] OR "myofibroblast"[Title/Abstract] OR "epithelial"[Title/Abstract] OR "endothelial"[Title/Abstract] OR "TGF-beta"[Title/Abstract] OR "extracellular matrix"[Title/Abstract] OR "senescence"[Title/Abstract] OR "glycolysis"[Title/Abstract] OR "epigenetic"[Title/Abstract] OR "clinical trial"[Title/Abstract] OR "randomized"[Title/Abstract])
